# Functionalization of anthracene: A selective route to brominated 1,4-anthraquinones

**DOI:** 10.3762/bjoc.7.118

**Published:** 2011-07-29

**Authors:** Kiymet Berkil Akar, Osman Cakmak, Orhan Büyükgüngör, Ertan Sahin

**Affiliations:** 1 Department of Chemistry, Faculty of Art and Science, Gaziosmanpasa University, 60250, Tokat, Turkey; 2 Department of Physics, Faculty of Art and Science, Ondokuzmayıs University, 55060, Samsun, Turkey; 3 Department of Chemistry, Faculty of Art and Science, Atatürk University, 25240, Erzurum, Turkey

**Keywords:** anthracene derivatives, anthracene-1,4-dione, aromatization, bromination, bromoanthracene, methoxyanthracene, silver-induced substitution

## Abstract

Efficient and stereoselective syntheses are described for the preparation of 2,3,9,10-tetrabromo-1,4-dimethoxy-1,2,3,4-tetrahydroanthracenes **7**, **8** and the corresponding 1,4-diol **17** by silver ion-assisted solvolysis of hexabromotetrahydroanthracene **6**. Base-promoted aromatization of **7** and **8** afforded synthetically valuable tribromo-1-methoxyanthracenes **10** and **11**. The reaction of **17** with sodium methoxide generated tribromodihydroanthracene-1,4-diol **27**, whose oxidation with PCC gave 2,9,10-tribromoanthracene-1,4-dione (**28**). Therefore a selective and efficient method was developed for the preparation of compound **28** starting from 9,10-dibromoanthracene (**1**), in a simple four-step process. Compounds **10** and **11**, and diol **27** constitute key precursors for the preparation of functionalized substituted anthracene derivatives that are difficult to prepare by other routes. The studies also reveal the broad range of reactivity and selectivity of the stereoisomeric anthracene derivatives.

## Introduction

**Scheme 1:**
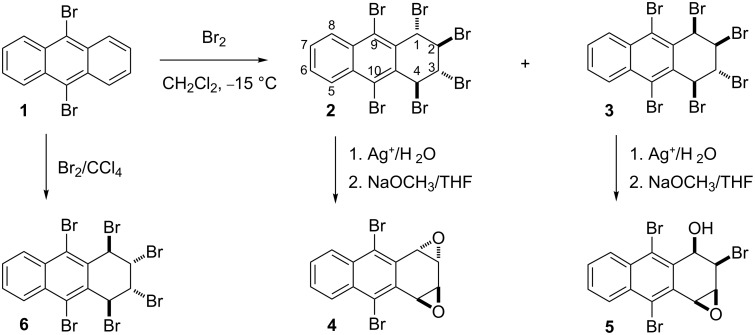
Synthesis of anthracene oxides.

Our sustained interest in benzenoid aromatic compounds with high bromine content has led to the development of a regio- and stereoselective bromination method for aromatic compounds. Recently, we demonstrated the selective bromination of 9,10-dibromoanthracene (**1**) to give hexabromides **2** and **3** (Scheme 1) [1]. These studies revealed that hexabromides **2** and **3** are good precursors for the preparation of anthracene oxides and methoxyanthracene derivatives by silver ion-induced substitution.

Our previous studies revealed that aromatization of hexabromides **2** and **3** showed complete selectivity both in their aromatization and silver-assisted solvolysis. On the other hand, most recently, we have developed an efficient stereoselective method to prepare hexabromide **6** [2–3] and opened up an efficient synthetic strategy for the synthesis of 3,9,10-tribromoanthracenes [2]. As an extension of this work, we report on new methoxy and hydroxy derivatives of anthracene, whose further transformation generates synthetically important novel anthracene derivatives. We also report an effective synthetic route to 1,4-dione **28** starting from 9,10-dibromoanthracene (**1**) in four steps. The compounds constitute potentially important keys for the synthesis of pharmaceutical chemicals [4–7], natural products [8–9] and donor properties [10–11]. Anthraquinone **28** may be used as precursor for 9,10-disubstituted-2-phenoxy-anthracene-1,4-dione derivatives that are difficult to synthesize by other routes. There has been much interest in the preparation of phenoxy-1,4-anthraquinone derivatives due to their anti-trypanosomal activity. Bolognesi et al. designed and synthesized a small library of 2-phenoxy-1,4-anthraquinone derivatives, all of which showed inhibitory activity toward either *Trypanosoma* or *Leishmania* species [12].

## Results and Discussion

**Scheme 2:**
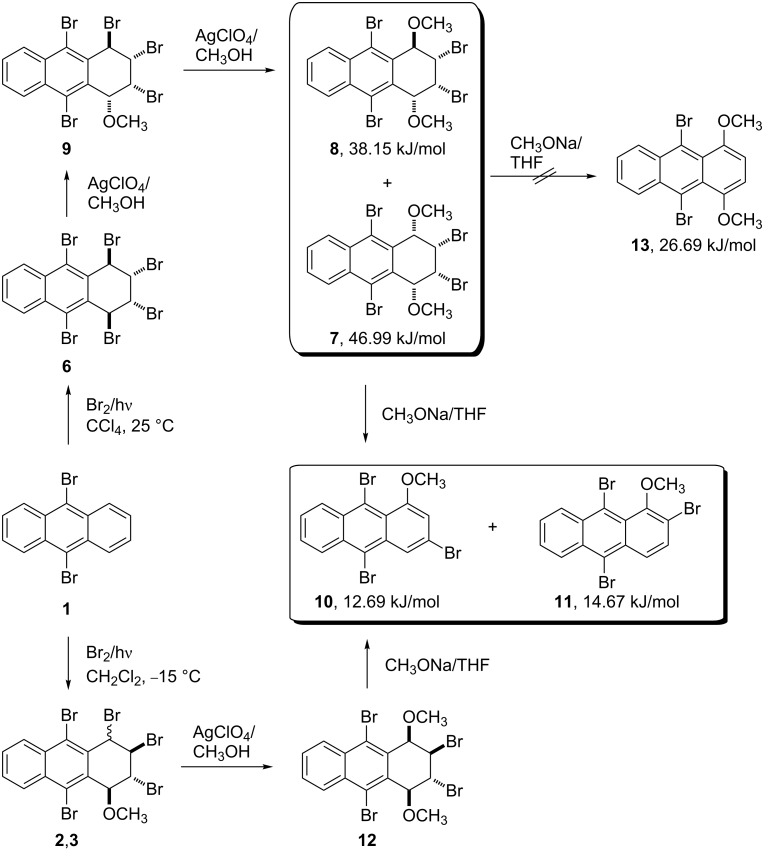
Synthesis of methoxyanthracenes **10** and **11**.

A solution of hexabromide **6** in dry methanol was treated with two equivalents of silver perchlorate at room temperature for two days in the dark. The reaction resulted in the formation of dimethoxy compounds **7** and **8** in a ratio of 37:63 as assigned by ^1^H NMR (Scheme 2). Although the methanolysis was repeated several times, the product ratio remained almost the same. The compounds were separable by column chromatography and were isolated in 29 and 44% yield for **7** and **8**, respectively.

**Figure 1:**
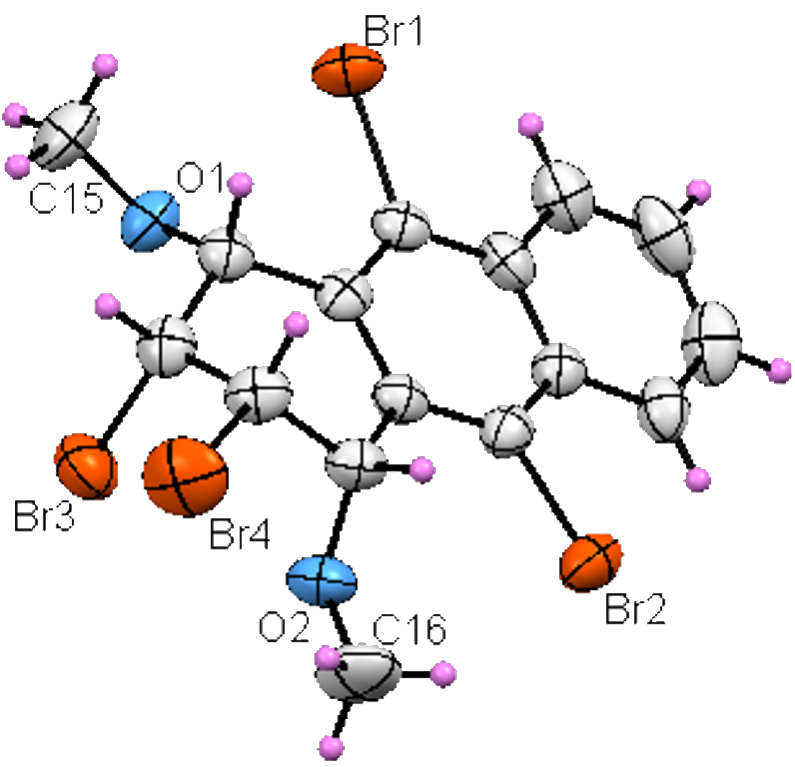
Molecular structure of compound **7**. Displacement ellipsoids are shown at 40% probability level.

There are three possible stereoisomers that can be formed in the reaction, however, it was difficult to distinguish the exact stereochemistry of symmetrical compounds **7** and **8** by NMR. Therefore, X-ray diffraction analysis was also carried out for **7**. The study confirmed the structure of **7** without any ambiguity (Figure 1).

Proton and carbon NMR studies indicated that tetrabromide **8** has an asymmetric structure. There is only one possible isomeric structure available for asymmetric dimethoxides. In the NMR spectrum of compound **8** all of the signals are multiplets, except for the two methoxy groups that appear as singlets (δ 4.05 and 3.66) and the doublet of H1 (*J* = 2.4 Hz). Resonances of aryl protons (δ 8.45 ppm for H5 and H8; δ 7.66 ppm for H6 and H7) are in accordance with the suggested structure. The aliphatic protons appear to be at δ 5.38 (d, *J* = 2.4 Hz, H1), δ 5.15 (m, H4 and H2) and δ 4.83 (m, H3), respectively. In particular, a 15-line (in fact 16, one of which is overlapped) ^13^C NMR spectrum supports the asymmetry in the molecule.

Formation of both, **7** and **8** can be explained from the initially formed product **9**. In the second step, a silver-promoted S_N_1 reaction of **9** results in the two compounds. Compound **7**, with all four substituents on the same face of the ring, possesses increased steric energy, which explains why compound **7** is obtained as minor isomer (Scheme 2).

After successful isolation of **7** and **8**, which both have the required skeletal arrangement and functionality to permit the easy introduction of two double bonds, the compounds were subjected to base-induced elimination. In separate reactions, both aromatizations afforded the same product mixture, i.e., 1-methoxy-3,9,10-tribromoanthracene (**10**) and 1-methoxy-2,9,10-tribromoanthracene (**11**), but in different ratios. The ratios of compounds **10** and **11** are 64:36 from **7** and 45:55 from **8**, respectively (Scheme 2). The compounds were easily separated by column chromatography and are readily crystallizable materials. Structure assignment for compounds **10** and **11** was carried out by comparison with authentic compounds [1].

**Scheme 3:**
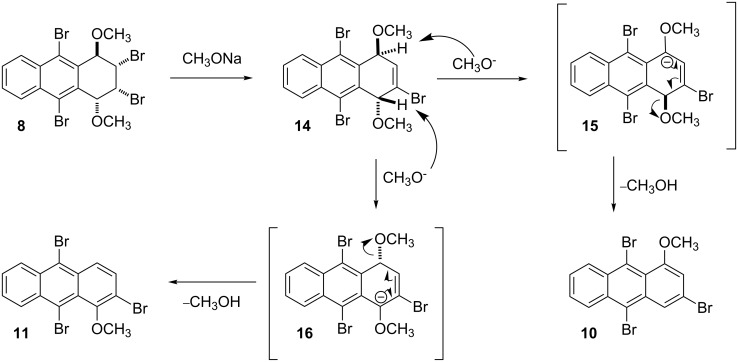
The reaction mechanism for the formation of methoxyanthracenes **10** and **11**.

To elucidate the mechanistic details and to explore the origin of selectivity, we also investigated the reaction of 1,4-dimethoxy compound **8** with one equivalent of sodium methoxide (Scheme 3). The reaction afforded a mixture of **14**, **11** and **10** in a ratio of 56:13:1, as calculated by integration of the ^1^H NMR signals of the methoxy groups, besides the remaining starting material (70% conversion). After chromatography, the major product **14** was isolated in 41% yield. The ^1^H NMR spectrum of **14** consisted of seven signal groups. An AA'BB'-like signal system of aryl protons appeared at δ 8.48 and δ 7.70. While the resonance of H1 was at 5.82 ppm as a singlet, the resonance of H4 at 5.44 ppm appeared as a doublet of doublet (*J* = 4 Hz and *J*_1,4_ = 2 Hz). The resonance of olefinic H3 was observed at 6.86 ppm as a doublet (*J* = 4.0 Hz). The singlets of methoxy signals were at δ 3.23 and δ 2.88. The ^13^C NMR spectrum of the compound consisted of twelve signals of aryl and double bond carbons; two sp^3^ carbons with methoxy groups (δ 76.0 and δ 74.4) and two methoxy carbons (δ 53.9 and δ 50.9).

An unusual mechanism for the formation of **14** from **8** is summarized in Scheme 3, which indicates that the base attacks on both H1 and H4 in tribromide **14** were followed by an elimination to provide compounds **10** and **11**. The formation of compound **13**, by elimination of two mol of HBr, was prevented, probably due to the enormously increased strain energy between the bulky groups of methoxy and bromine at the γ*-*gauche positions, C1/C4 and C9/C10, respectively (Scheme 2) [13] . Additionally, the single crystal X-ray crystallographic data for **7** illustrates the sterically crowded environment of H1 and H4, in which base attachment is hindered, and which shows the axial-like orientation of H1 and H4 and the equatorial-like orientation of methoxy groups in C1 and C4. Therefore, we assume that proper diaxial configuration of H2 and Br3 in compounds **7** and **8** induces E2 elimination to give compound **14** (Figure 1).

**Scheme 4:**
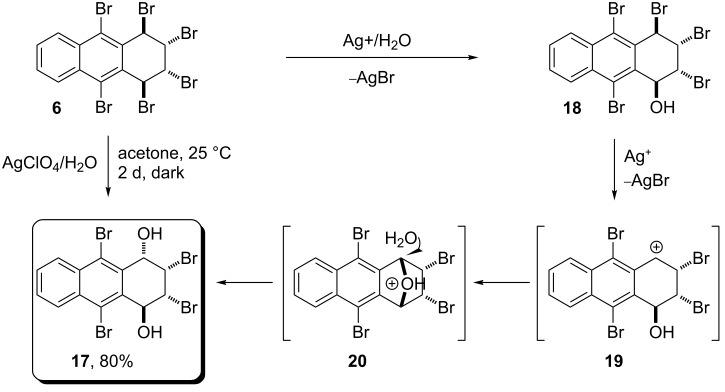
The formation mechanism for dihydroxy **17**.

For further functionalization of the anthracene skeleton, hexabromide **6** was subjected to silver ion-assisted hydrolysis in aqueous acetone. Interestingly, the substitution resulted in the stereoselective formation of product **17** in 80% yield (Scheme 4). Mass spectral analysis of compound **17** confirmed the molecular signal at *m*/*z* 413 and the ^1^H NMR spectrum indicates its asymmetry. The ^13^C NMR spectrum also confirms the asymmetric structure consisting of ten sp^2^ carbon signals (six quaternary and four methine carbons) and four sp^3^ carbon signals. It is obvious, that there is only one possible asymmetric isomeric structure of **17** due to the retention of the configuration of the bromines on the C2- and C3-atoms. H1 and H4 protons can be easily identified due to their coupling with the hydroxy groups (*J*_1,OH_ = 4.0; *J*_4,OH_ = 8.4 Hz). A comparison of the chemical shifts and coupling constants with the products obtained in previous studies [1] is helpful for the identification of isomeric structures. The resonances of benzylic protons (H1 and H4) shift to lower fields than those of H2 and H3, as shown in our previous similar examples.

In the proton NMR spectrum, the signals at δ 5.90 and δ 5.44 were assigned to H1 and H4, respectively. The resonance of H4 is a doublet of a doublet at δ 5.44 (m). Therefore the doublet at δ 3.10 belongs to C4-OH (*J*_4,OH_ = 8.4 Hz). H1 resonates as a multiplet at δ 5.90 due to the nearly identical coupling constant value (*J*_1,OH_ = 4 Hz). The hydroxy group (C1-OH) appears at δ 2.90 as a doublet (*J* ≈ 4 Hz). Finally, H2 and H3 resonate at δ 4.79 and δ 5.26 (*J* << 4 Hz), respectively. The resonances of H5 and H8 at δ 8.49 and δ 8.41 are two different multiplets, while those of H6 and H7 are a multiplet at δ 7.71.

**Figure 2:**
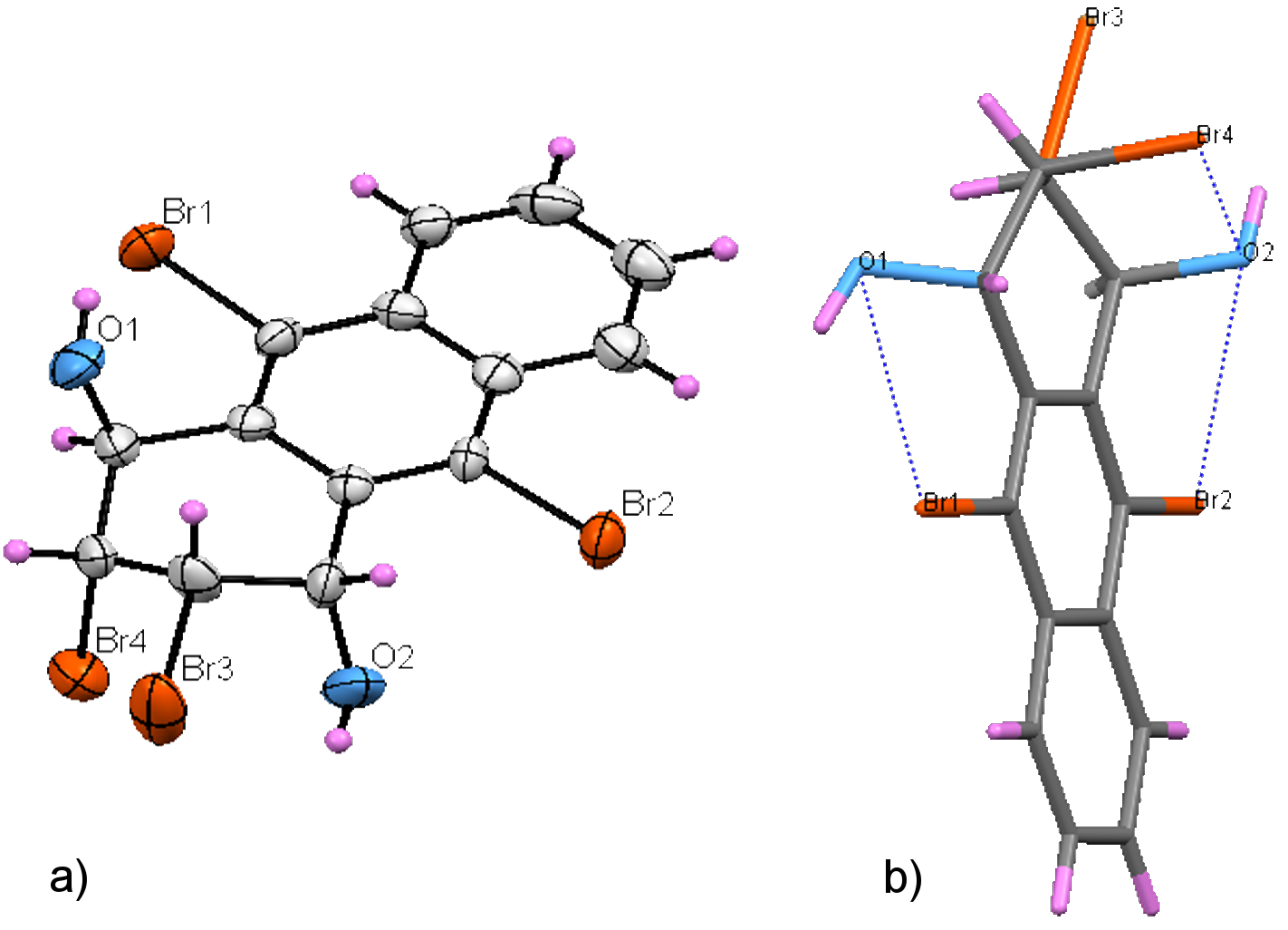
a) X-ray ORTEP plot of compound **17**. Displacement ellipsoids are shown at 40% probability level. b) X-ray structure of **17** showing intramolecular interactions.

Finally, X-ray analysis of the compound was performed and the exact configuration was proved to be dihydroxy **17** (Figure 2). The stereoselective formation of **17** can be explained by the neighboring-group participation, as shown in Scheme 4. We assume that *cis* orientation of bromine atoms in C2 and C3 induces the formation of a bridged-oxonium ion **20** as an intermediate of **17**.

**Scheme 5:**
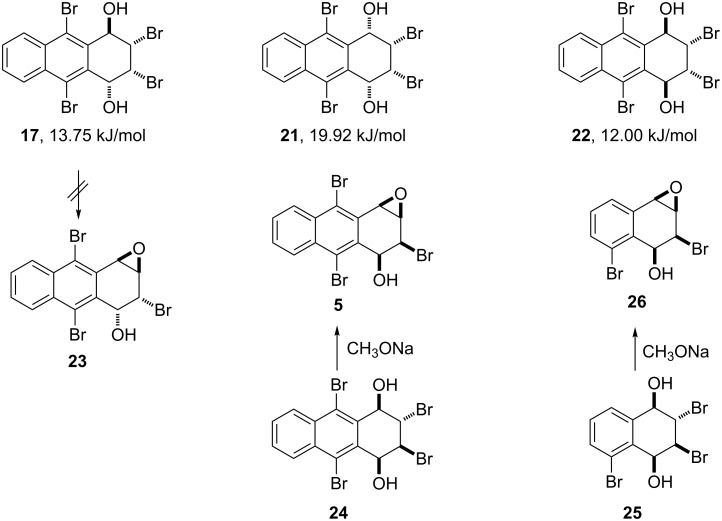
Base-promoted reaction of the dihydroxides and formation of the epoxides.

**Scheme 6:**
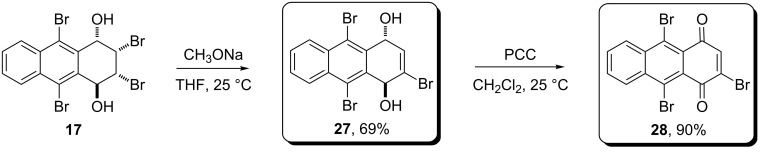
Synthesis of compounds **27** and **28**.

Hydrolysis of hexabromide **6** can lead to three halohydrines (**17**, **21** and **22)**, two of which are symmetric (Scheme 5). Dihalohydrine **17** is expected to be a good precursor to corresponding arene oxides **23** as shown in our previous studies (Scheme 1 and Scheme 5) [1,14–16]. Therefore, one or two equivalents of sodium methoxide were applied to **17** and the reaction resulted in the formation of **27** contrary to the expected epoxide **23** or any aromatized products (Scheme 6). In our earlier study, the X-ray packing structure of tetralin oxide **26** produced from the corresponding halohydrine **25**, shows the existence of hydrogen bonding between the hydroxy and the epoxy group, which has the same stereochemistry as that of compound **5** [1,14–15]. Thus, the fact that compound **23** was not obtained from **17** can be attributed to the absence of hydrogen bonding due to the *anti*-orientation of the substituents in **23**, although compound **23** is energetically more favourable than the corresponding compound **5**.

The structure of compound **27** was determined by MS, IR, and ^1^H and ^13^C NMR. The mass spectrum of compound **27** gave a [M − 2OH] ^+^ peak at *m*/*z* 413 corresponding to the formula and the IR spectrum showed a hydroxy band at 3540 and 3338 cm^−1^. The ^1^H NMR spectrum consists of simple signal systems; two aryl signals as multiplets at δ 8.42 for H5 and H8, and at δ 7.72 for H6 and H7, and an olefinic signal at δ 6.69 (*J* = 4.4 Hz) as a doublet. In addition, the aliphatic signals H1 and H4 appear at δ 5.80 (d, *J*_1,OH_ = 6 Hz) and δ 5.74 (dd, *J* = 4.4 Hz, *J*_4,OH_ = 6 Hz), respectively. The hydroxy protons resonate at δ 3.28 and δ 3.22 as doublets with the same coupling constants. The ^13^C NMR spectrum with twelve signals is also consistent with the structure of the compound.

It is clear that 1,4-diol **27** should be easily converted to anthracene-1,4-dione **28**. Thus, compound **27** was made to react with PCC at room temperature for 3 days. After the reaction, a simple short silica gel filtration and crystallization from CH_2_Cl_2_, gave the expected 2,9,10-tribromoanthracene-1,4-dione (**28**) in high yield (90%) (Scheme 6). The ^1^H NMR spectrum of the compound consisted of four signal systems, one of which is a singlet (8.81, H3), while the others are multiplets at 8.74 (H8), 8.48 (H5) and 7.84 (H6 and H7). The ^13^C NMR spectrum confirms the structure with no aliphatic carbon but two carbonyl signals at δ 177.0 and δ 174.3, respectively.

## Conclusion

This report and our previous studies [1–2] demonstrate that the reactivity and selectivity of the stereoisomeric anthracene derivatives strongly depend on both, the reaction conditions and their stereochemistry. For example, although both hydroxy compounds obtained from hexabromides **2** and **3** [1] afforded epoxides **4** and **5** upon treatment with sodium methoxide (Scheme 1, Scheme 5), dihydroxy compound **17** produced olefinic compound **27**, whose oxidation gave diketone **28**. We also observed different reactivities towards the base for the hydroxy and the methoxy compounds. For example, the methoxy compounds aromatize to give **10** and **11**, but the diols convert either to the epoxides by cyclization (Scheme 1 and Scheme 5) or to the alkene by elimination of 1 mol HBr (Scheme 6). In addition, however, the base-induced reaction of both the methoxy stereoisomers **7** and **8**, generated from hexabromide **6**, and of methoxy compound **12**, obtained from hexabromide **2** and **3**, gave the same compounds **10** and **11** by elimination of one mol of HBr and CH_3_OH.

In conclusion, a convenient and effective procedure for the synthesis of dimethoxy **7** and **8** was developed, where base-mediated elimination yields the synthetically valuable 1-methoxy-3,9,10-tribromoanthracene (**10**) and 1-methoxy-2,9,10- tribromoanthracene (**11**), which are very useful precursors for the synthesis of many other substituted anthracenes due to the easy substitution of the bromo substituents. It is noteworthy that separation and crystallization of methoxylated bromoanthracenes is relatively easy compared to bromoanthracenes [1], and this offers important advantages for preparative purposes. Additionally, we describe the first isolation and identification of an anthracene-1,4-dione **28** starting from 1,2,3,4,9,10-hexabromo-1,2,3,4-tetrahydroanthracene **6**. The most important advantage is the complete selectivity of each reaction step. Although the reactions described follow simple synthetic methodology, the results are quite appealing. The present procedure is particularly suitable for the preparation of poly-substituted anthracene-1,4-dione and pentacene derivatives, in which the bromo substituents enable further functionalization.

As a consequence, the studies revealed that the reactivity of the anthracene derivatives displays interesting selectivity and appears to provide a simple route to a range of highly functionalized and potentially useful polysubstituted anthracenes.

## Experimental

**General:** Thin layer chromatography was carried out on Merck 0.255 mm silica gel F_254_ analytical aluminum plates and spots were visualized with UV fluorescence at 254 nm. Classic gravity column chromatography was performed using silica gel 60 (70–230 mesh, Merck). Melting points were determined on a Thomas-Hoover capillary melting point apparatus. Solvents were concentrated at reduced pressure. Infrared spectra were obtained from KBr pellets on a Jasco FT/IR 430 instrument. Mass spectra were recorded on an Agilent 6890 GC System 5973 MSD spectrometer and THERMO FINNIGAN spectrometer under electron impact (EI) conditions. NMR spectra were recorded on a Bruker spectrometer at 400 MHz for ^1^H and at 100 MHz for ^13^C NMR.

### Reaction of hexabromide **6** with **2** equiv of silver perchlorate

To a stirred solution of hexabromide **6** (600 mg, 0.915 mmol) in dry and freshly distilled methanol (60 mL) was added silver perchlorate (475 mg, 2.287 mmol) under an argon atmosphere in the dark. The resulting reaction mixture was stirred magnetically at rt for 2 d. Reaction progress was monitored by TLC for consumption of the starting material. The silver bromide precipitate was removed by filtration and to the mother liquor was added methylene chloride (50 mL). The material was washed with water (4 × 50 mL) and dried over Na_2_SO_4_. After drying and removal of the solvent, NMR analysis showed two compounds in a ratio of 63:37. The residue (374 mg) was purified by column chromatography on silica gel (20 g) using *n*-hexane as eluent. The first fraction was (1R*,2R*,3S*,4R*)-2,3,9,10-tetrabromo-1,4-dimethoxy-1,2,3,4-tetrahydroanthracene (**8**): (224 mg, 44%, colorless crystals). The second fraction was (1R*,2R*,3S*,4S*)-2,3,9,10-tetrabromo-1,4-dimethoxy-1,2,3,4-tetrahydroanthracene (**7**) (144 mg, 29%, colorless crystals).

(1R*,2R*,3S*,4R*)-2,3,9,10-Tetrabromo-1,4-dimethoxy-1,2,3,4-tetrahydroanthracene (8) mp 160–162 °C (dichloromethane/hexane); ^1^H NMR (400 MHz, CDCl_3_) δ 8.45 (m, 2H), 7.66 (m, 2H), 5.38 (d, 1H, *J* = 2.4 Hz), 5.15 (m, 2H), 4.83 (m,1H), 4.05 (s, 3H), 3.66 (s, 3H); ^13^C NMR (100 MHz, CDCl_3_) δ 134.1, 133.4, 133.3, 130.6, 129.0, 128.9, 128.5, 128.3, 128.2, 83.5, 79.9, 64.6, 58.9, 48.8, 48.7; IR (KBr, cm^−1^): 2978, 2927, 2824, 1567, 1540, 1521, 1506, 1478, 1455, 1439, 1404, 1371, 1353, 1331, 1275, 1250, 1331, 1201, 1181, 1158, 1131, 1079, 1067, 972, 935, 916, 862, 836, 762, 753, 691, 660, 620, 602, 570, 541, 478, 458; MS (APCI) *m*/*z* 576.3 [M + NH_4_]^+^, 560.1 [M + 2H]^+^, 526.6 [M − 2OCH_3_]^+^, 475.7 [M − Br − 2H]^+^, 445.7 [M − OCH_3_ – Br + H] ^+^, 395.8 [M − 2Br − 2H]^+^, 365.8 [M − OCH_3_ − 2Br]^+^, 335.8 [M − 2OCH_3_ − 2Br]^+^, 312.2, 298.3, 284.2, 270.2, 256.1, 242.2, 228.2, 84.1; Anal. calcd for C_16_H_14_Br_4_O_2_: C, 34.45; H, 2.53; found: C, 34.43; H, 2.562.

(1R*,2R*,3S*,4S*)*-*2,3,9,10-Tetrabromo-1,4-dimethoxy-1,2,3,4-tetrahydroanthracene (7) mp 153–154 °C (dichloromethane/hexane); ^1^H NMR (400 MHz, CDCl_3_) δ 8.45 (m, 2H), 7.69 (m, 2H), 5.16 (m, 2H), 4.78 (m, 2H), 3.75 (s, 6H); ^13^C NMR (100 MHz, CDCl_3_) δ 133.4, 133.2, 129.0, 128.5, 127.3, 79.9, 59.8, 51.2; IR (KBr, cm^−1^): 2949, 2921, 2826, 1698, 1671, 1559, 1478, 1438, 1348, 1333, 1276, 1249, 1186, 1161, 1123, 1078, 1002, 973, 941, 911, 860, 831, 776, 761, 728, 677, 629, 610, 582, 526, 507, 473; MS (APCI) *m*/*z* 526.6 [M − OCH_3_]^+^, 494.6 [M − 2OCH_3_ − H]^+^, 446.7 [M − OCH_3_ − Br]^+^, 413.7 [M − 2OCH_3_ − Br − 2H]^+^, 396.9 [M − 2Br − H]^+^, 365.8 [M − OCH_3_ − 2Br]^+^, 335.9 [M − 2OCH_3_ − 2Br]^+^, 316.0, 303.0, 279.1, 152.0, 79.1, 65.2; Anal. calcd for C_16_H_14_Br_4_O_2_: C, 34.45; H, 2.53; found: C, 35.57; H, 2.59.

### Aromatization of compound **7**

A stirred solution of compound **7** (150 mg, 0.27 mmol) in dry and freshly distilled THF (20 mL) was combined with a solution of sodium methoxide (44 mg, 0.806 mmol) in freshly distilled THF (15 mL) under an argon atmosphere. The resulting reaction mixture was magnetically stirred for 12 h at 25 °C. Ether (40 mL) was added to the reaction mixture and the resulting precipitate was washed with water (3 × 50 mL) and dried over anhydrous sodium sulfate. The solvent was evaporated. ^1^H NMR of the residue (0.105 g, total yield: 90%) showed two products in a ratio of 64:36 for **11** and **10**, respectively. The residue (0.105 g) was purified by column chromatography on silica gel (40 g) using *n*-hexane as eluent. The first fraction was 1-methoxy-3,9,10-tribromoanthracene (**10**) (22 mg, 19%, yellow crystals). The second fraction was 1-methoxy-2,9,10-tribromoanthracene (**11**) (51 mg, 43%, yellow crystals).

**1-Methoxy-3,9,10-tribromoanthracene (10)** mp 159–160 °C (chloroform/hexane); ^1^H NMR (400 MHz, CDCl_3_) δ 8.80 (m, 1H), 8.39 (m, 1H), 8.30 (d, *J*_2,4_ = 1.7 Hz, 1H), 7.50 (m, 2H), 6.83 (d, *J*_2,4_ = 1.7 Hz, 1H), 3.93 (s, 3H); ^13^C NMR (100 MHz, CDCl_3_) δ 156.7, 133.1, 132.2, 131.6, 129.4, 128.4, 128.2, 127.6, 123.1, 122.9, 122.4, 121.5, 119.4, 109.5, 55.9; IR (KBr, cm^−1^): 2956, 2925, 2854, 1618, 1596, 1540, 1523, 1457, 1427, 1373, 1349, 1303, 1267, 1247, 1093, 983, 923, 885, 833, 815, 811,744, 549, 437, 410; MS (GC-MS) *m*/*z* 443 [M − 2H]^+^, 430 [M − OCH_3_]^+^, 400, 364 [M − Br]^+^, 350, 322, 283 [M − 2Br]^+^, 255, 240, 222, 204 [M − 3Br]^+^, 189, 173 [M − OCH_3_ − Br]^+^, 160, 148, 134, 127, 121, 86, 80, 73, 67, 61, 27, 17; Anal. calcd for C_15_H_9_Br_3_O: C, 40.49; H, 2.04; found C, 40.40; H, 2.08.

**1-Methoxy-2,9,10-tribromoanthracene (11)** mp 164–165 °C (dichloromethane/hexane); ^1^H NMR (400 MHz, CDCl_3_) δ 8.86 (m, 1H), 8.57 (m, 1H), 8.38 (d, *J* = 9.6 Hz, 1H), 7.71 (d, *J* = 9.6 Hz, 1H), 7.68 (m, 2H), 3.91 (s, 3H); ^13^C NMR (100 MHz, CDCl_3_) δ 152.8, 132.8, 131.8, 131.3, 131.2, 129.1, 128.4, 128.3, 128.1, 126.7, 126.1, 124.8, 117.4, 116.8, 61.8; IR (KBr, cm^−1^): 2991, 2976, 2931, 2834, 1698, 1670, 1652, 1617, 1591, 1539, 1515, 1446, 1427, 1375, 1332, 1291, 1251, 1144, 1057, 1032, 966, 929, 905, 796, 763, 744, 706, 651, 549, 532, 505, 481; Anal. calcd for C_15_H_9_Br_3_O: C, 40.49; H, 2.04; found: C, 40.08; H, 2.15.

### Aromatization of compound **8**

A stirred solution of compound **8** (300 mg, 0.54 mmol) in dry and freshly distilled THF (30 mL) was combined with a solution of sodium methoxide (73 mg, 1.34 mmol) in freshly distilled THF (20 mL) under an argon atmosphere. The resulting reaction mixture was magnetically stirred for 12 h at 25 °C. Ether (50 mL) was added to the reaction mixture and the resulting precipitate was washed with water (3 × 50 mL) and dried over anhydrous sodium sulfate. The solvent was evaporated. ^1^H NMR of the residue (217 mg, total yield: 91%) showed two products in a ratio of 45:55 for **10** and **11**, respectively.

### The reaction of compound **8** with one equivalent of sodium methoxide

To a stirred solution of compound **8** (200 mg, 0.36 mmol) in dry and freshly distilled THF (40 mL) was added sodium methoxide (21 mg, 0.394 mmol) under argon atmosphere. The resulting reaction mixture was magnetically stirred for 1 d at 25 °C. Diethyl ether (30 mL) was added to the reaction mixture and the resulting precipitate was washed with water (3 × 30 mL) and dried over anhydrous sodium sulfate. The solvent was evaporated. ^1^H NMR of the residue (132 mg) showed three products in a ratio of 56:13:1 for **14**, **11** and **10**, respectively, besides the remaining starting material **8** (conversion 70%). The mixture was separated by thin layer chromatography to give pure compounds (SiO_2_, hexane). Compound **14** was obtained in a yield of 14% (50 mg).

(1R*,4S*)*-*2,9,10-Tribromo-1,4-dimethoxy-1,4-dihydroanthracene (14) mp 135–136 °C (dichloromethane/hexane); ^1^H NMR (400 MHz, CDCl_3_) δ 8.48 (m, 2H), 7.70 (m, 2H), 6.86 (d, *J* = 4 Hz, 1H), 5.82 (s, 1H), 5.44 (dd, *J* = 4 Hz, *J*_1,4_ = 2 Hz, 1H), 3.23 (s, 3H), 2.88 (s, 3H); ^13^C NMR (100 MHz, CDCl_3_) δ 133.2, 133.1, 132.8, 132.5, 131.6, 128.9, 129.0, 128.4, 128.2, 126.2, 125.8, 124.3, 76.0, 74.4, 53.9, 50.9; IR (KBr, cm^−1^): 3056, 2989, 2931, 2823, 1670, 1563, 1479, 1457, 1438, 1344, 1305, 1267, 1251, 1170, 1037, 946, 921, 900, 852, 755, 701, 651, 644, 624, 597, 572, 553, 514, 453, 426; MS (APCI) *m*/*z* 468.0 [M − OCH_3_ + Na − H]^+^, 444.9 [M − OCH_3_ − H]^+^, 422.1 [M – Br + Na]^+^, 395.0 [M − Br]^+^, 366.1 [M − 2Br + 2Na + 3H]^+^, 329 [M − OCH_3_ − 2Br + 2Na − 2H]^+^, 227.1; Anal. calcd for C_16_H_13_Br_3_O_2_: C, 40.29; H, 2.75; found: C, 40.37; H, 2.82.

### Hydrolysis of hexabromide **6**

To a stirred solution of hexabromide **6** (1000 mg, 1.52 mmol) in acetone (100 mL) was added a solution of AgClO_4_·H_2_O (696 mg, 3.35 mmol) in aqueous acetone (6 mL acetone + 7 mL water) in the dark. The resulting mixture was stirred at rt for 2 d in the dark. After the reaction was complete (TLC), the AgBr precipitate was filtered off and the reaction mixture was diluted with methylene chloride (30 mL). The organic layer was washed with H_2_O (3 × 30 mL) and dried over Na_2_SO_4_. After removal of the solvent, the precipitate (0.698 g) was crystallized from methanol (15 mL). (1S*,2R*,3S*,4S*)-2,3,9,10-tetrabromo-1,2,3,4-tetrahydroanthracene-1,4-diol (**17**) was obtained in a yield of 80% (641 mg).

(1S*,2R*,3S*,4S*)-2,3,9,10-Tetrabromo-1,2,3,4-tetrahydroanthracene-1,4-diol (17) mp 178–180 °C; ^1^H NMR (400 MHz, CDCl_3_) δ 8.49 (m, 1H), 8.41 (m, 1H), 7.71 (m, 2H), 5.90 (m, 1H), 5.44 (m, 1H), 5.26 (m, 1H), 4.79 (m, 1H), 3.10 (d, *J*_4,OH_ = 8.4 Hz, 1H), 2.90 (d, *J*_1,OH_ = 4 Hz, 1H); ^13^C NMR (100 MHz, CDCl_3_) δ 133.6, 133.4, 133.1, 131.6, 129.3, 129.2, 128.8, 128.5, 128.0, 127.3, 74.6, 69.9, 53.2, 48.9; IR (KBr, cm^−1^): 3509, 3405, 3068, 2983, 2931, 2904, 2825, 1716, 1617, 1567, 1479, 1444, 1407, 1334, 1247, 1180, 1157, 1135, 1097, 1039, 981, 929, 896, 877, 811, 786, 759, 690, 647, 636, 619, 601, 570, 538, 474, 441; MS (GC-MS/EI) *m*/*z* 413 [M − 2OH − Br − 2H]^+^, 334 [M − 2OH − 2Br − H]^+^, 255 [M − 2OH − 3Br]^+^, 206, 173 [M − 2OH − 4Br − 2H]^+^, 166, 148, 134, 127, 121, 109, 97, 87, 73, 61, 49, 38, 30, 27, 17; Anal. calcd for C_14_H_10_Br_4_O_2_: C, 31.74; H, 1.90; found C, 32.45; H, 2.01.

### Synthesis of 2,9,10-tribromo-1,4-dihydroanthracene-1,4-diol (**27**)

(1S*,2R*,3S*,4S*)-2,3,9,10-Tetrabromo-1,2,3,4-tetrahydroanthracene-1,4-diol (**17**) (475 mg, 0.896 mmol) in dry THF (20 mL) was treated with sodium methoxide (53.35 mg, 0.95 mmol) in dry THF (15 mL). The mixture was stirred at rt overnight. After the reaction was complete (TLC), the reaction was diluted with diethyl ether (20 mL) and washed with H_2_O (3 × 20 mL). The organic layer was dried over anhydrous sodium sulfate and concentrated under reduced pressure. After the residue was passed through a short Al_2_O_3_ (neutral, 15 g) column, 2,9,10-tribromo-1,4-dihydroanthracene-1,4-diol (**27**) was recrystallized from methylene chloride/hexane (20:5 mL), yield 276 mg (69%). The reaction was repeated with two equiv of NaOMe (106.7 mg, 1.9 mmol) under the same conditions and identical results were obtained.

**2,9,10-Tribromo-1,4-dihydroanhracene-1,4-diol (27)** mp 141–142 °C (dichloromethane/hexane); ^1^H NMR (400 MHz, CDCl_3_) δ 8.42 (m, 2H), 7.72 (m, 2H), 6.69 (d, *J* = 4.4 Hz, 1H), 5.80 (d, *J* = 6 Hz, 1H), 5.74 (dd, 1H), 3.28 (d, *J*_1,OH_ = 6 Hz, 1H), 3.22 (1H, d, *J*_4,OH_ = 6 Hz); ^13^C NMR (100 MHz, CDCl_3_) δ 134.3, 132.9, 132.6, 131.3, 129.1, 129.1, 128.6, 128.1, 127.8, 125.8, 125.7, 125.5, 70.2, 67.5; IR (KBr, cm^−1^): 3540, 3338, 3064, 2952, 2873, 2840, 1673, 1567, 1479, 1403, 1344, 1311, 1247, 1166, 1118, 1051, 975, 900, 856, 835, 752, 700, 647, 545, 455; MS (GC-MS/EI) *m*/*z* 413 [M − 2OH]^+^, 334 [M − 2OH − Br]^+^, 253 [M − 2OH − 2Br]^+^, 206, 173 [M − 2OH − 3Br]^+^, 166, 148, 134, 127, 121, 109, 97, 86, 73, 61, 49, 38, 30, 28, 17; Anal. calcd for C_14_H_9_Br_3_O_2_: C, 37.46; H, 2.02; found: C, 37.57; H, 2.28.

### Synthesis of 2,9,10-tribromoanthracene-1,4-dione (**28**)

To a solution of pyridinium chlorochromate (300 mg, 1.08 mmol) in methylene chloride (30 mL) was added a solution of 2,9,10-tribromo-1,4-dihydroanthracene-1,4-diol (**27**) (222 mg, 0.504 mmol) in methylene chloride (25 mL). The mixture was stirred at ambient temperature for 3 d. Reaction progress was monitored by TLC for consumption of the starting material. After the residue was filtered through a short silica gel (15 g) column eluting with dichloromethane (200 mL), 2,9,10-tribromoanthracene-1,4-dione (**28**) was crystallized from methylene chloride/hexane (2:1), yield 198 mg (90%).

**2,9,10-Tribromoanthracene-1,4-dione (28)** mp 251.5–252.5 °C; ^1^H NMR (400 MHz, CDCl_3_) δ 8.81 (s, 1H), 8.74 (m, 1H), 8.48 (m, 1H), 7.84 (m, 2H); ^13^C NMR (100 MHz, CDCl_3_) δ 177.0, 174.3, 146.1, 134.7, 134.1, 132.5, 131.8, 131.6, 131.2, 130.3, 130.0, 129.7, 128.1, 124.3; IR (KBr, cm^−1^): 3056, 2921, 2850, 1679, 1600, 1536, 1475, 1440, 1380, 1369, 1346, 1319, 1294, 1251, 1230, 1222, 1189, 1162, 1020, 1004, 919, 875, 862, 757, 701, 669, 601,566, 549, 420; MS (APCI) *m*/*z* 489.1 [M + Na + NH_4_ + 3H]^+^, 467.9 [M + Na]^+^, 446.0 [M + H]^+^, 421.1, 409.0 [M – Br + NH_4_ + Na + 3H]^+^, 386.1 [M – Br + NH_4_ + 3H]^+^, 355.0, 329.0, 310.2 [M − 2Br + Na + 2H]^+^, 271.0, 254.0, 227.1 [M − 3Br + Na + H]^+^, 149.1; Anal. Calcd for C_14_H_5_Br_3_O_2_: C, 37.79; H, 1.13; found: C, 37.79; H, 1.31.

### Crystallography

For crystal structure determination, single crystals of compounds **7** and **17** were used for data collection on a four-circle Rigaku R-AXIS RAPID-S diffractometer (equipped with a two-dimensional area IP detector). The graphite-monochromated Mo Kα radiation (λ = 0.71073 Å) and oscillation scan technique with Δω = 5° for one image were used for data collection. The lattice parameters were determined by the least-squares methods on the basis of all reflections with F^2^ > 2σ (F^2^). Integration of the intensities, correction for Lorentz and polarization effects and cell refinement was performed with CrystalClear software [17]. The structures were solved by direct methods with SHELXS-97 [18] and refined by a full-matrix least-squares procedure with the program SHELXL-97 [18]. Hydrogen positions were found from difference Fourier maps and geometrical calculations, and refined using a riding model. The final difference Fourier maps showed no peaks of chemical significance.

**Crystal data for 7:** C_16_H_14_Br_4_O_2_, crystal system, space group: Monoclinic, *P*2_1_/*c*; unit cell dimensions: *a* = 12.8322 (6) Å, *b* = 13.2320(7) Å, *c* = 11.1619(5) Å, α = 90°, β = 115.092(3)°, γ = 90°, volume: 1716.38(14) Å^3^; *Z* = 4; calculated density: 2.159 Mg/m^3^; absorption coefficient: 9.38 mm^–1^; F(000): 1064; θ-range for data collection 1.8–26.0°; refinement method: full-matrix least-square on *F*^2^; data/parameters: 2440/199; goodness-of-fit on *F*^2^: 1.527; final R indices [I > 2σ(I)]: R_1_ = 0.048, wR_2_ = 0.0843; R indices (all data): R_1_ = 0.0776, wR_2_ = 0.093; largest diff. peak and hole: 0.40 and –0.51 e Å^−3^; CCDC: 804575. **Crystal data for 17:** C_14_H_10_O_2_Br_4_, crystal system, space group: monoclinic, *P*2_1_/c ; unit cell dimensions: *a* = 7.9547(4), *b* = 11.9678(4), *c* = 15.5015(6) Å, α = 90°, β = 101.14(3)°, γ = 90˚; volume: 1447.92(2) Å^3^; *Z* = 4; calculated density: 2.43 Mg/m^3^; absorption coefficient: 11.113 mm^–1^; *F*(000): 1000; θ-range for data collection 2.6–26.4˚; refinement method: full-matrix least-square on *F*^2^; data/parameters: 2168/183; goodness-of-fit on *F*^2^: 1.217; final *R* indices [I > 2σ(I)]: *R*_1_ = 0.069, w*R*_2_ = 0.088; *R* indices (all data): *R*_1_ = 0.102, w*R*_2_ = 0.095; largest diff. peak and hole: 0.505 and –0.647 e Å^–3^; CCDC: 804289.

## Supporting Information

Supporting information features copies of ^1^H NMR and ^13^C NMR spectra for all new compounds and crystallographic information files for compounds **7** and **17**.

File 1 NMR spectra of compounds **7**, **8**, **10**, **11**, **14**, **17**, **27** and **28**.

File 2 Crystallographic information file for compound **7**.

File 3 Crystallographic information file for compound **17**.
