# Supplementary material for: Functionalization of anthracene: A selective route to brominated 1,4-anthraquinones
Source: Beilstein J Org Chem. 2011 Jul 29;7:1036–45. doi: 10.3762/bjoc.7.118 (PMC3167179; doi:10.3762/bjoc.7.118)
Supplement: File 1 — NMR spectra of compounds 7, 8, 10, 11, 14, 17, 27 and 28. [file Beilstein_J_Org_Chem-07-1036-s001.pdf]

Supporting Information

for

Functionalization of anthracene: A selective route to brominated 1,4-anthraquinones

Kiymet Berkil Akar[1], Osman Cakmak *[1], Orhan Büyükgüngör[2,§], Ertan Sahin[3,§]

Address: [1]Department of Chemistry, Faculty of Art and Science, Gaziosmanpasa University, 60250, Tokat, Turkey; [2]Department of Physics, Faculty of Art and Science, Ondokuzmayıs University, 55060, Samsun, Turkey and [3]Department of Chemistry, Faculty of Art and Science, Atatürk University, 25240, Erzurum, Turkey

Email: Kiymet Berkil Akar - kiymetberkil@gmail.com,*Osman Cakmak- cakmak.osman@gmail.com, Orhan Büyükgüngör - orhanb@omu.edu.tr, Ertan Sahin - ertan@atauni.edu.tr

*Corresponding author

[§] Authors to whom inquiries concerning the X-ray structure should be directed.

NMR spectra of compounds **7**, **8**, **10**, **11**, **14**, **17**, **27** and **28**

Content

^{1}H NMR spectrum of **7** S2
^{13}C NMR spectrum of **7** S3
^{1}H NMR spectrum of **8** S4
^{13}C NMR spectrum of **8** S5
^{1}H NMR spectrum of **10** S6
^{13}C NMR spectrum of **10** S7
^{1}H NMR spectrum of **11** S8
^{13}C NMR spectrum of **11** S9
^{1}H NMR spectrum of **14** S10
^{13}C NMR spectrum of **14** S11
^{1}H NMR spectrum of **17** S12
^{13}C NMR spectrum of **17** S13
^{1}H NMR spectrum of **27** S14
^{13}C NMR spectrum of **27** S15
^{1}H NMR spectrum of **28** S16
^{13}C NMR spectrum of **28** S17

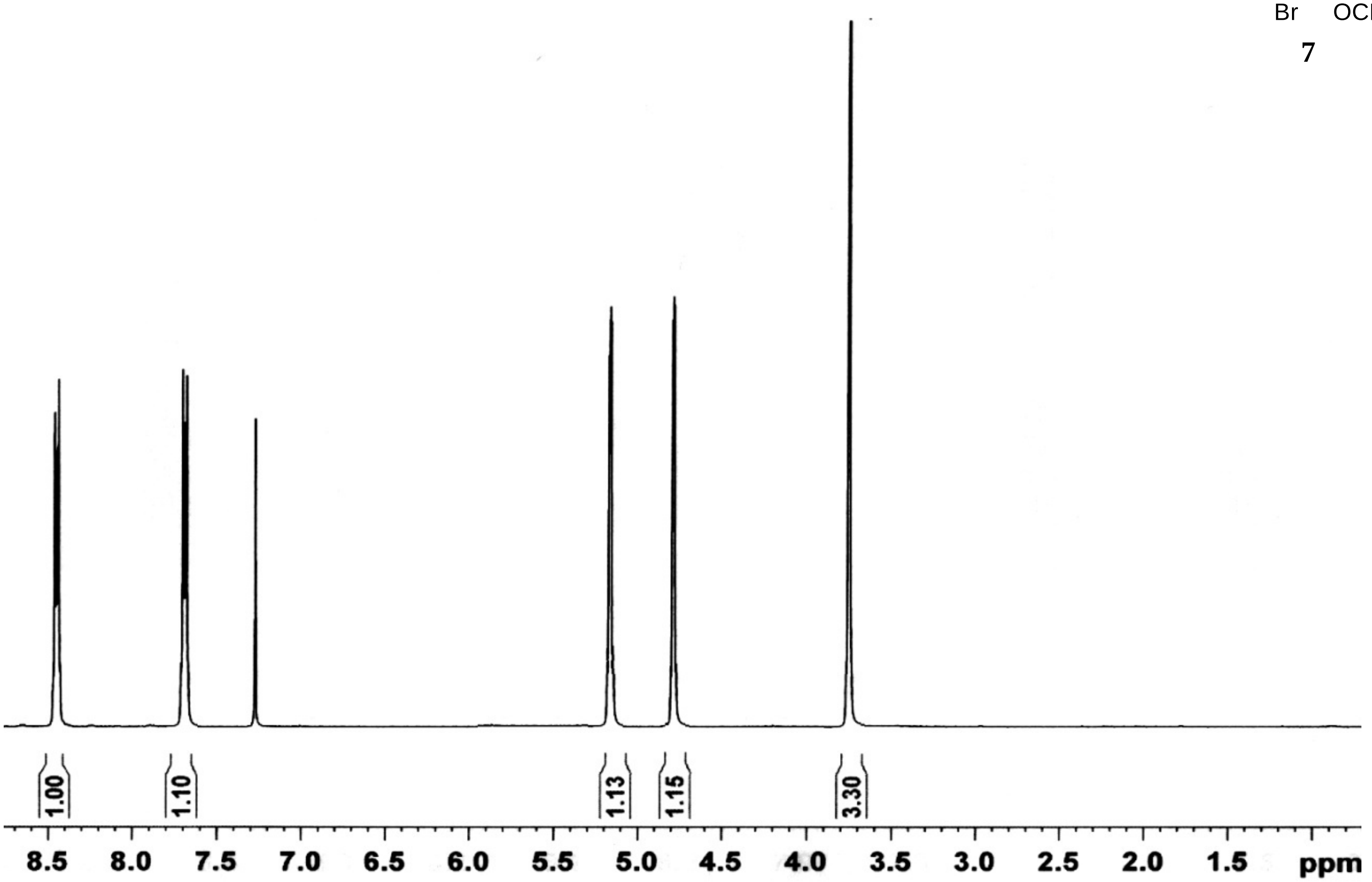
8.5
8.0
7.5
7.0
6.5
6.0
5.5
5.0
4.5
4.0
3.5
3.0
2.5
2.0
1.5
ppm
1.00
1.10
1.13
1.15
3.30

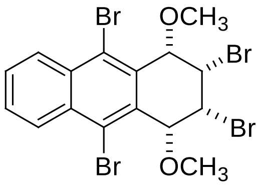
Br
OCH3
Br
Br
Br
OCH3

7

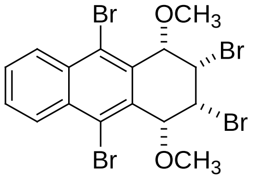
Br
OCH3
Br
Br
Br
OCH3
7

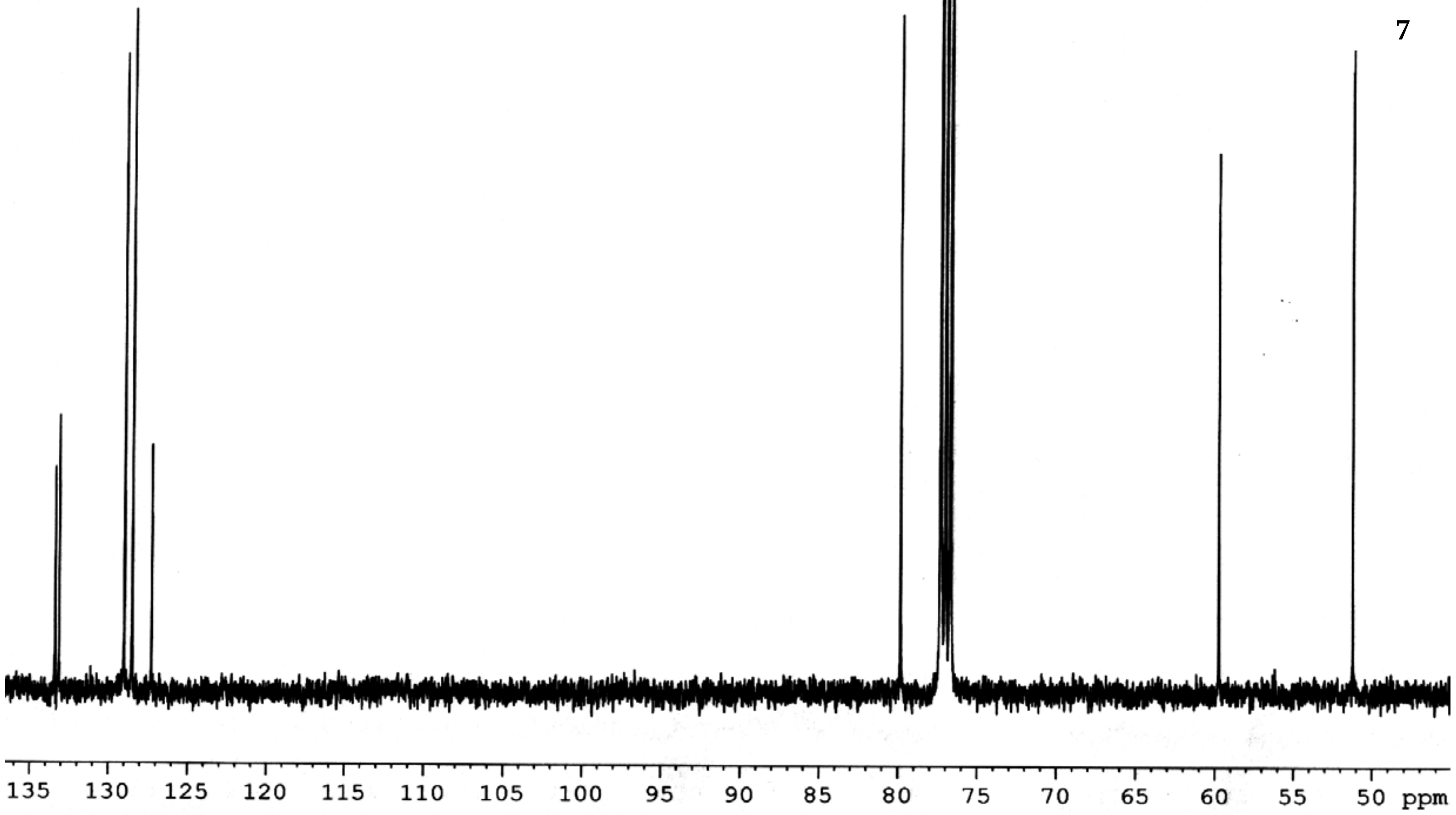
135 130 125 120 115 110 105 100 95 90 85 80 75 70 65 60 55 50 ppm

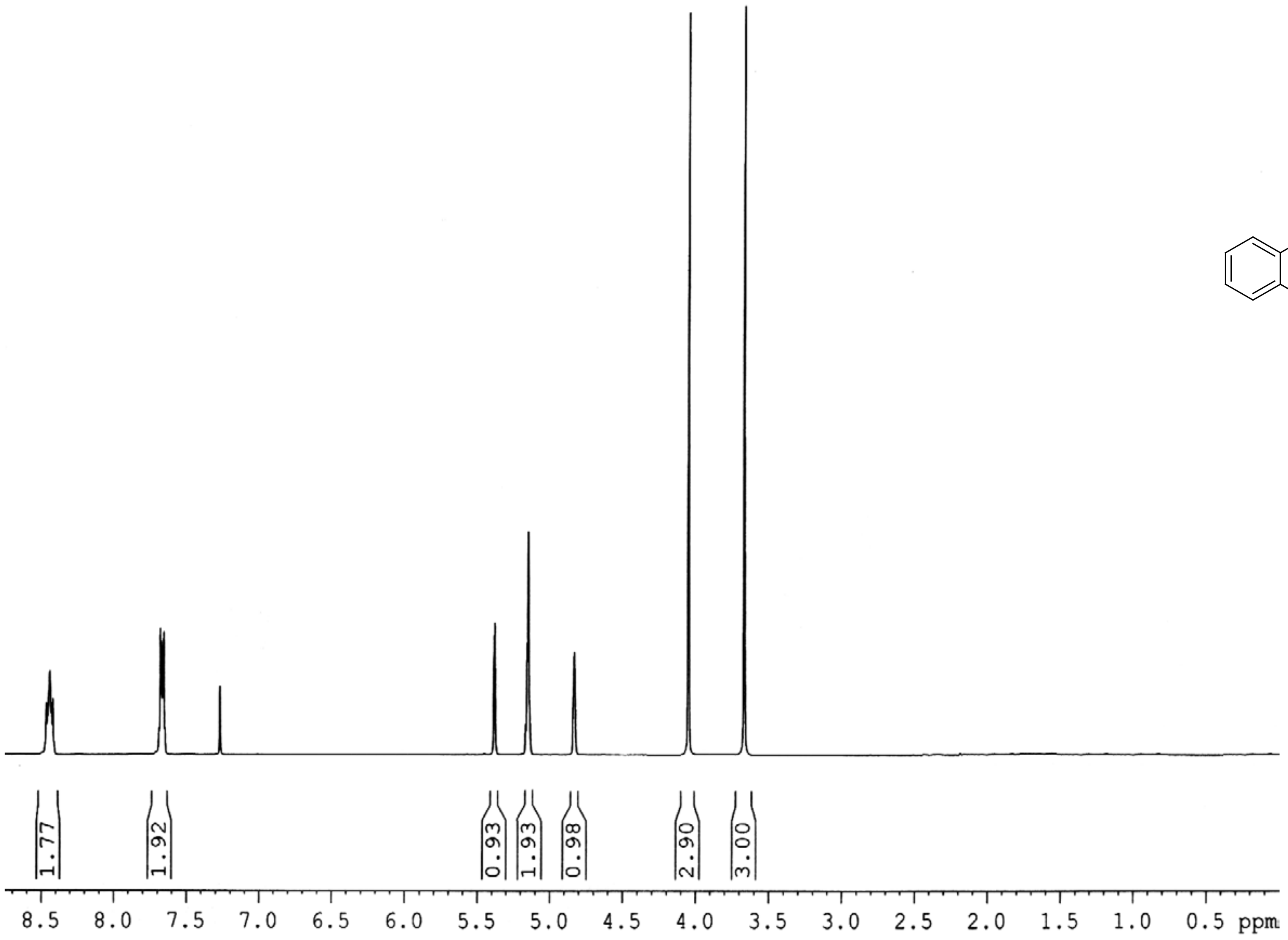
1.77
1.92
0.93
1.93
0.98
2.90
3.00
8.5 8.0 7.5 7.0 6.5 6.0 5.5 5.0 4.5 4.0 3.5 3.0 2.5 2.0 1.5 1.0 0.5 ppm

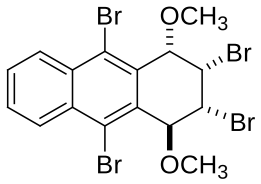
Br
OCH3
Br
Br
Br
OCH3

8

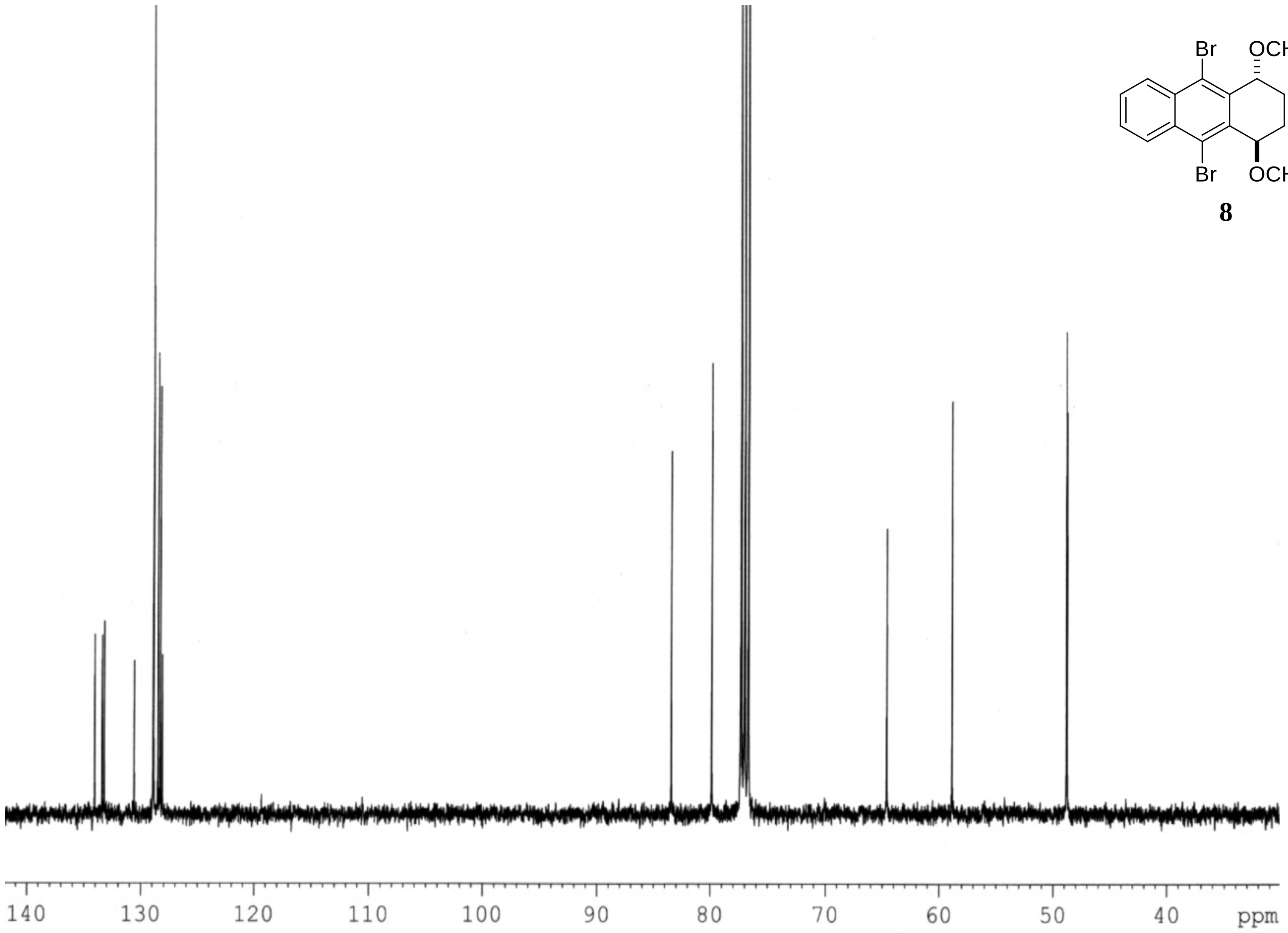
Br
OCH3
Br
Br
Br
OCH3
8
140
130
120
110
100
90
80
70
60
50
40
ppm

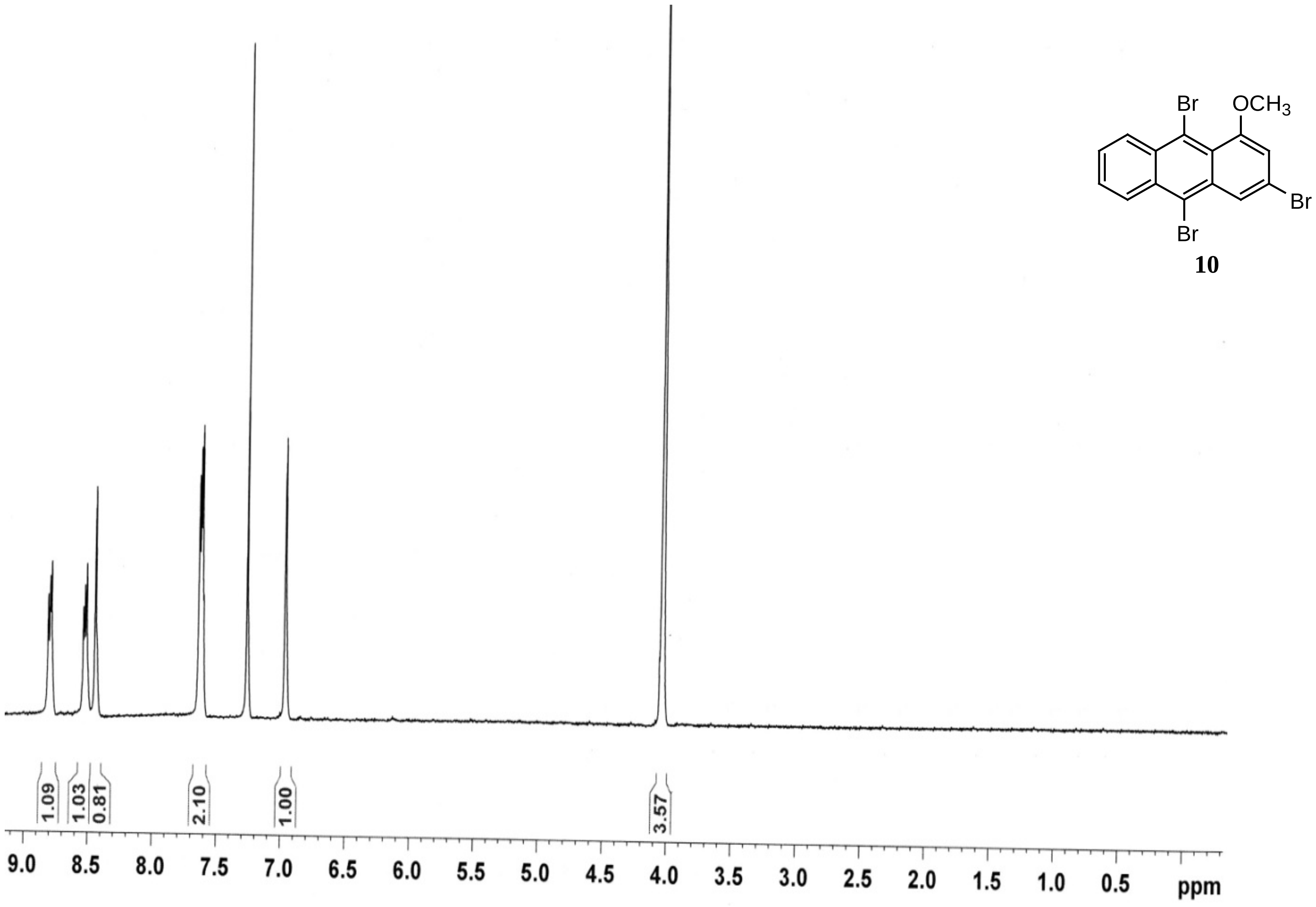
1.09
1.03
0.81
2.10
1.00
3.57
9.0
8.5
8.0
7.5
7.0
6.5
6.0
5.5
5.0
4.5
4.0
3.5
3.0
2.5
2.0
1.5
1.0
0.5
ppm
Br
OCH3
Br
Br

10

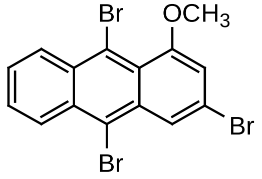

10

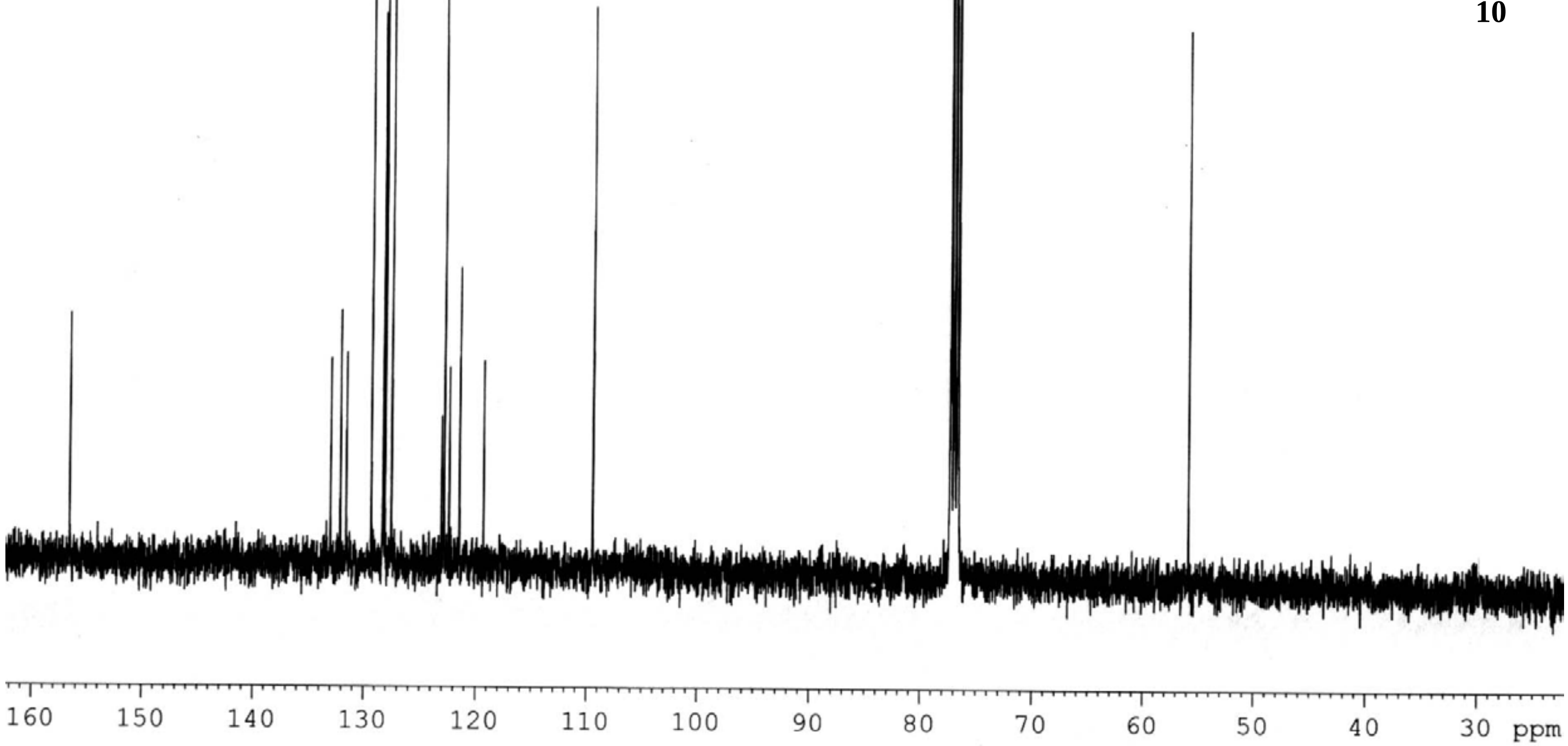

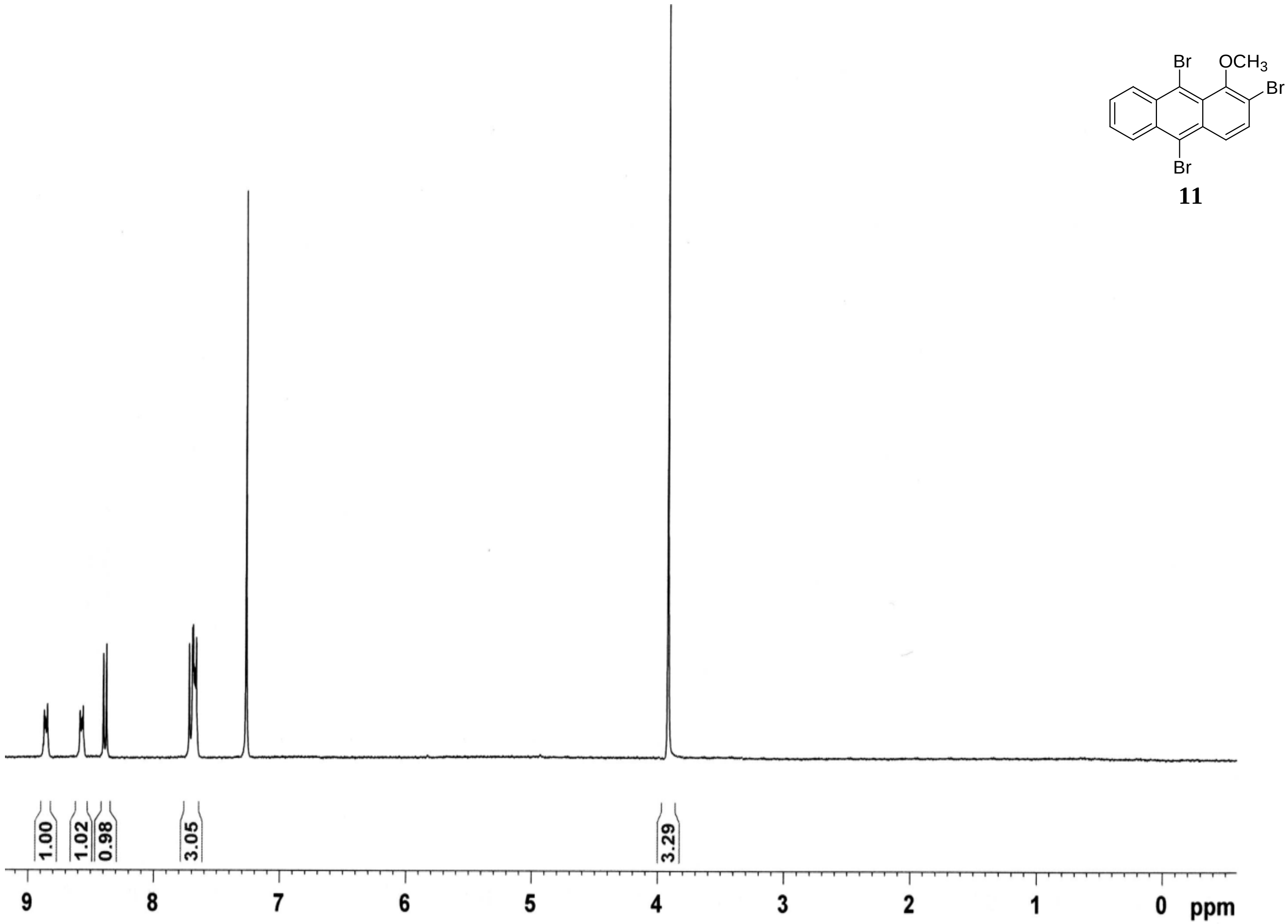

11

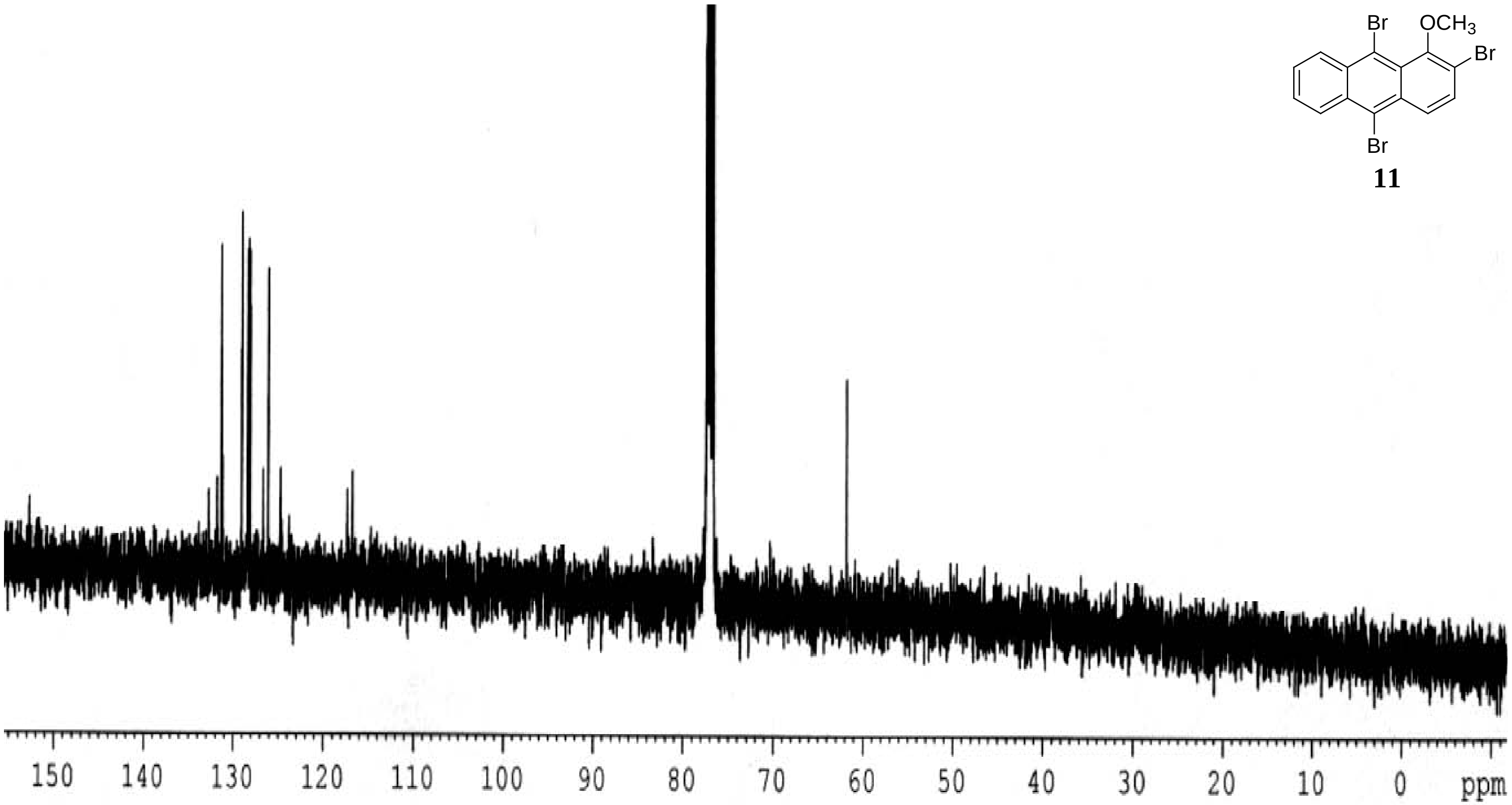
Br
OCH3
Br
Br
11
150 140 130 120 110 100 90 80 70 60 50 40 30 20 10 0 ppm

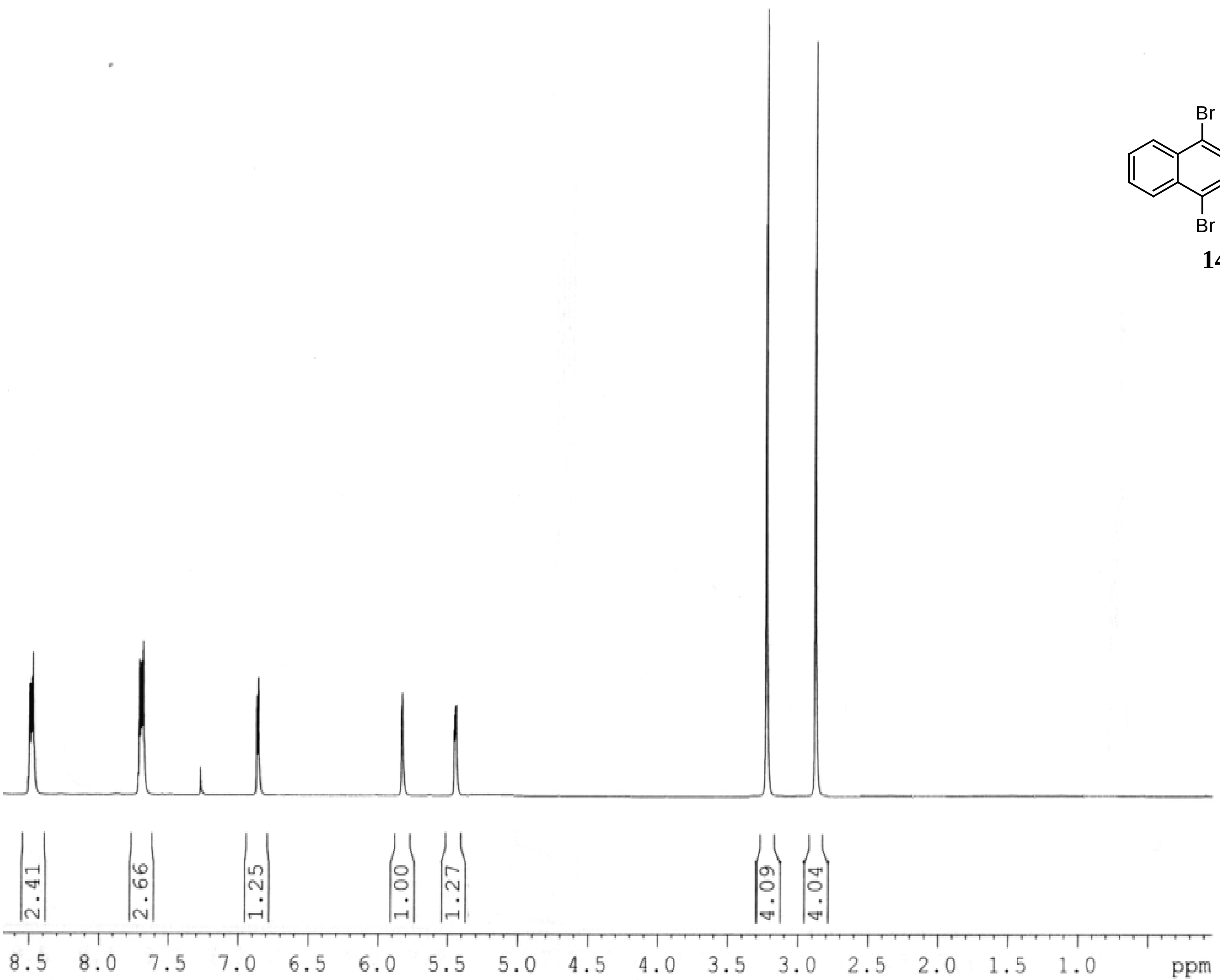
2.41
2.66
1.25
1.00
1.27
4.09
4.04
8.5 8.0 7.5 7.0 6.5 6.0 5.5 5.0 4.5 4.0 3.5 3.0 2.5 2.0 1.5 1.0
ppm

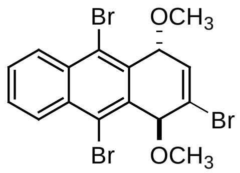
Br
OCH3
Br
Br
OCH3

14

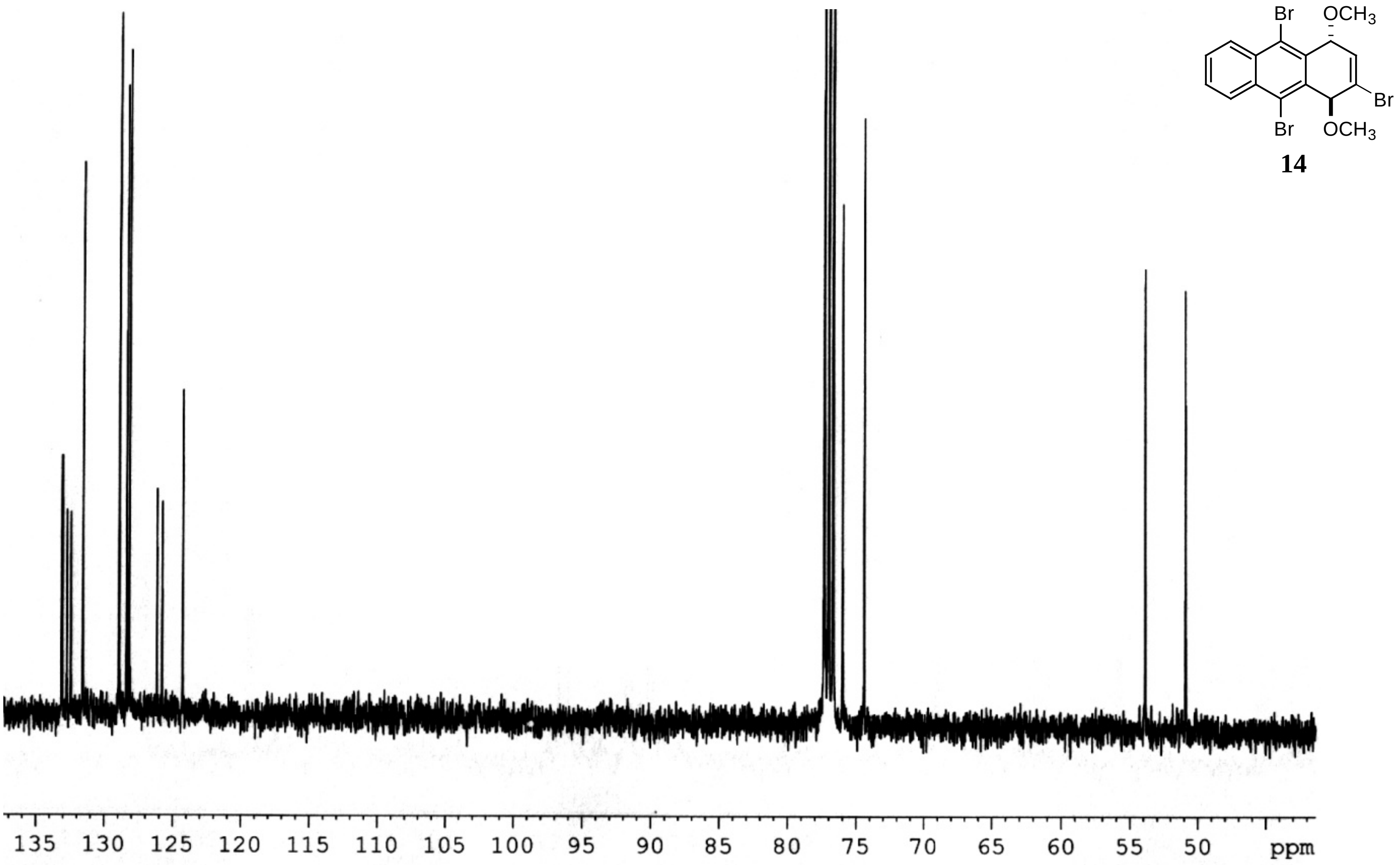
135
130
125
120
115
110
105
100
95
90
85
80
75
70
65
60
55
50
ppm
Br
OCH3
Br
Br
OCH3

14

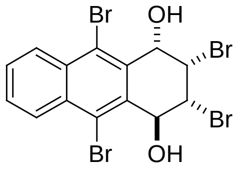
Br
OH
Br
Br
Br
OH
17

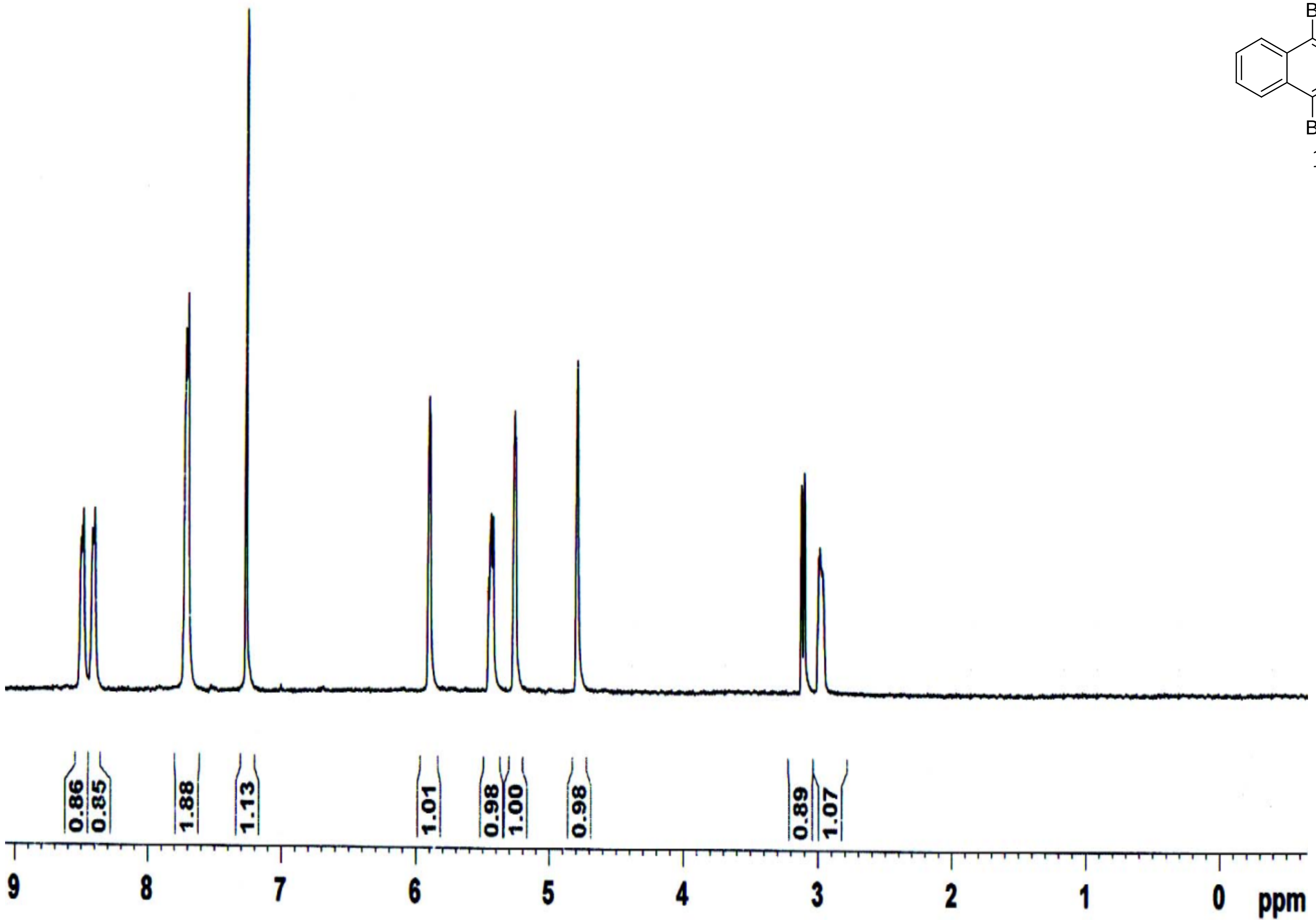
0.86
0.85
1.88
1.13
1.01
0.98
1.00
0.98
0.89
1.07
9
8
7
6
5
4
3
2
1
0
ppm

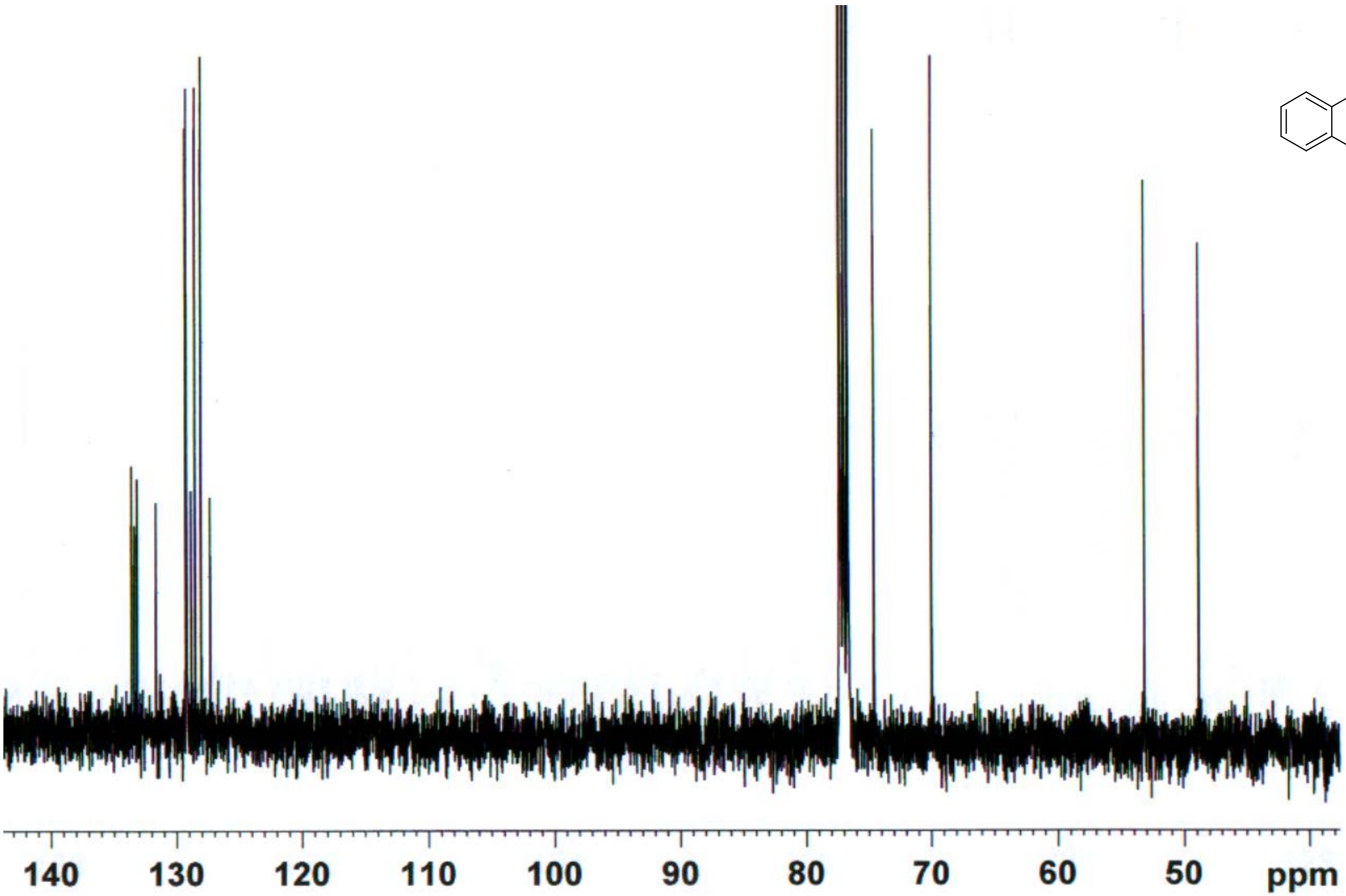

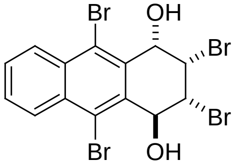

17

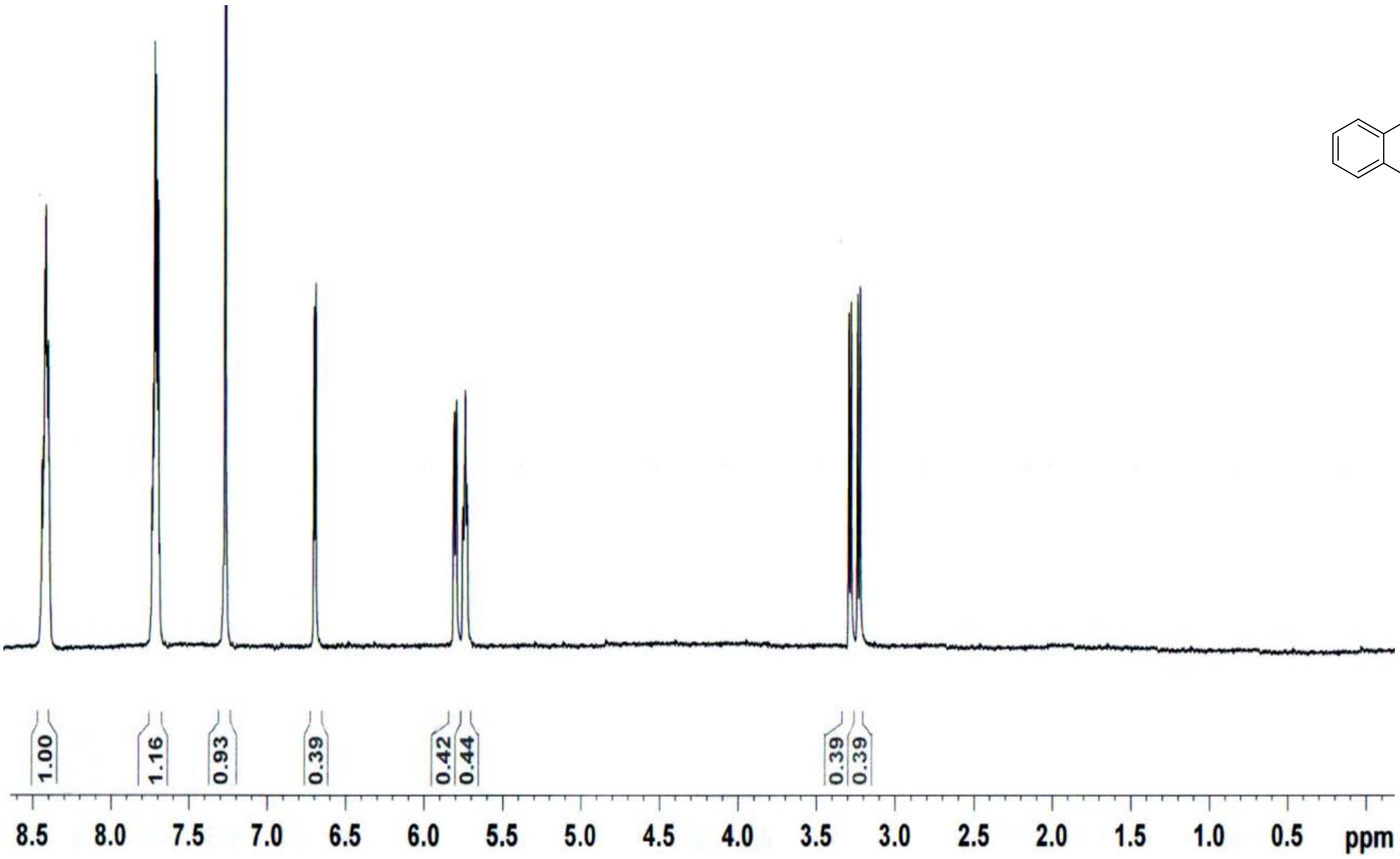
1.00
1.16
0.93
0.39
0.42
0.44
0.39
0.39
8.5 8.0 7.5 7.0 6.5 6.0 5.5 5.0 4.5 4.0 3.5 3.0 2.5 2.0 1.5 1.0 0.5 ppm

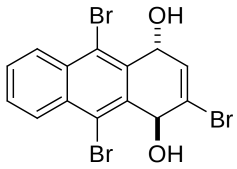
Br
OH
Br
Br
OH

27

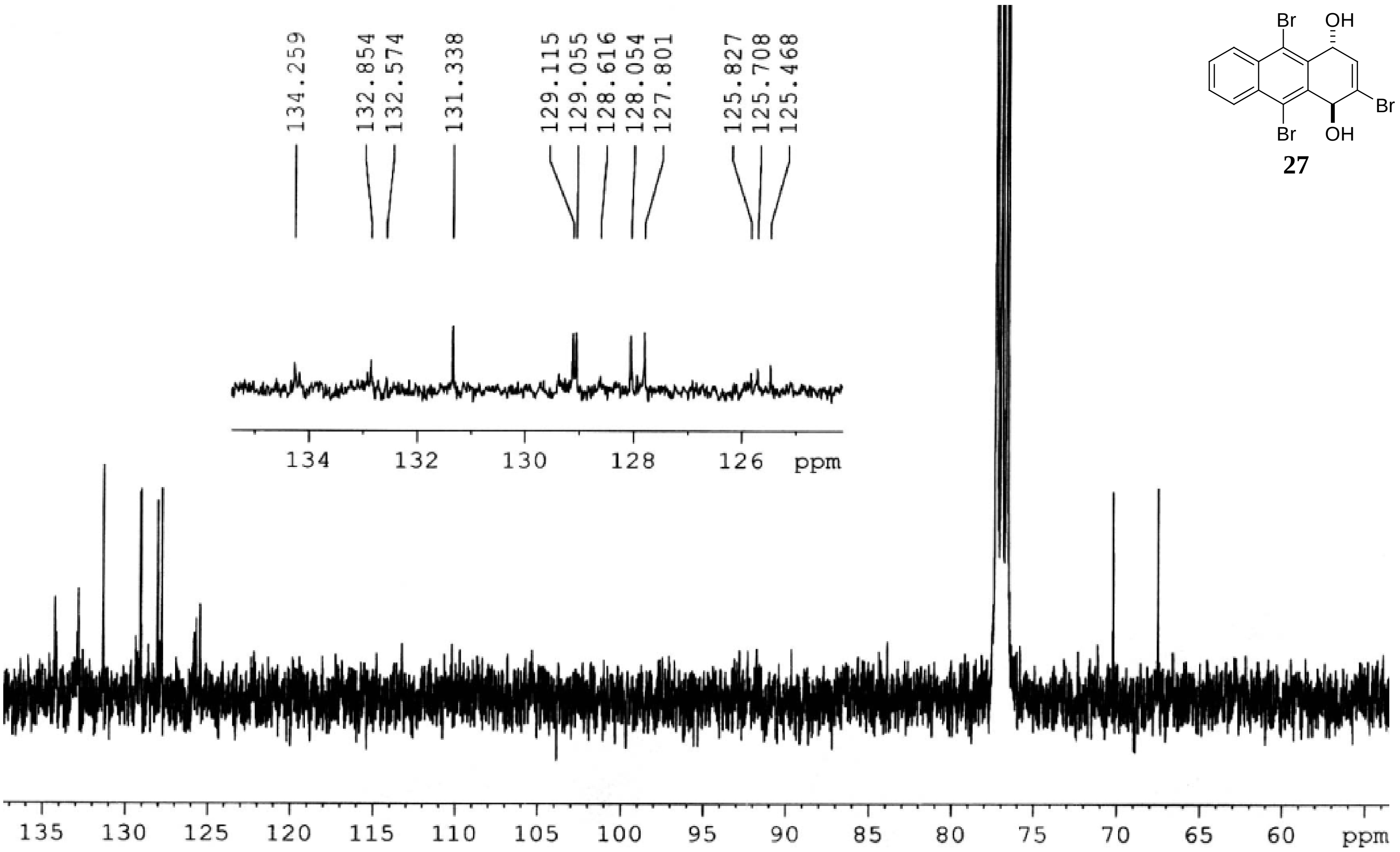

134.259
132.854
132.574
131.338
129.115
129.055
128.616
128.054
127.801
125.827
125.708
125.468
134 132 130 128 126 ppm
135 130 125 120 115 110 105 100 95 90 85 80 75 70 65 60 ppm
Br
OH
Br
Br
OH

27

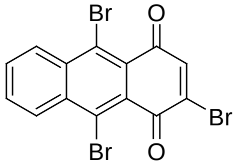
Br
O
Br
Br
O

28

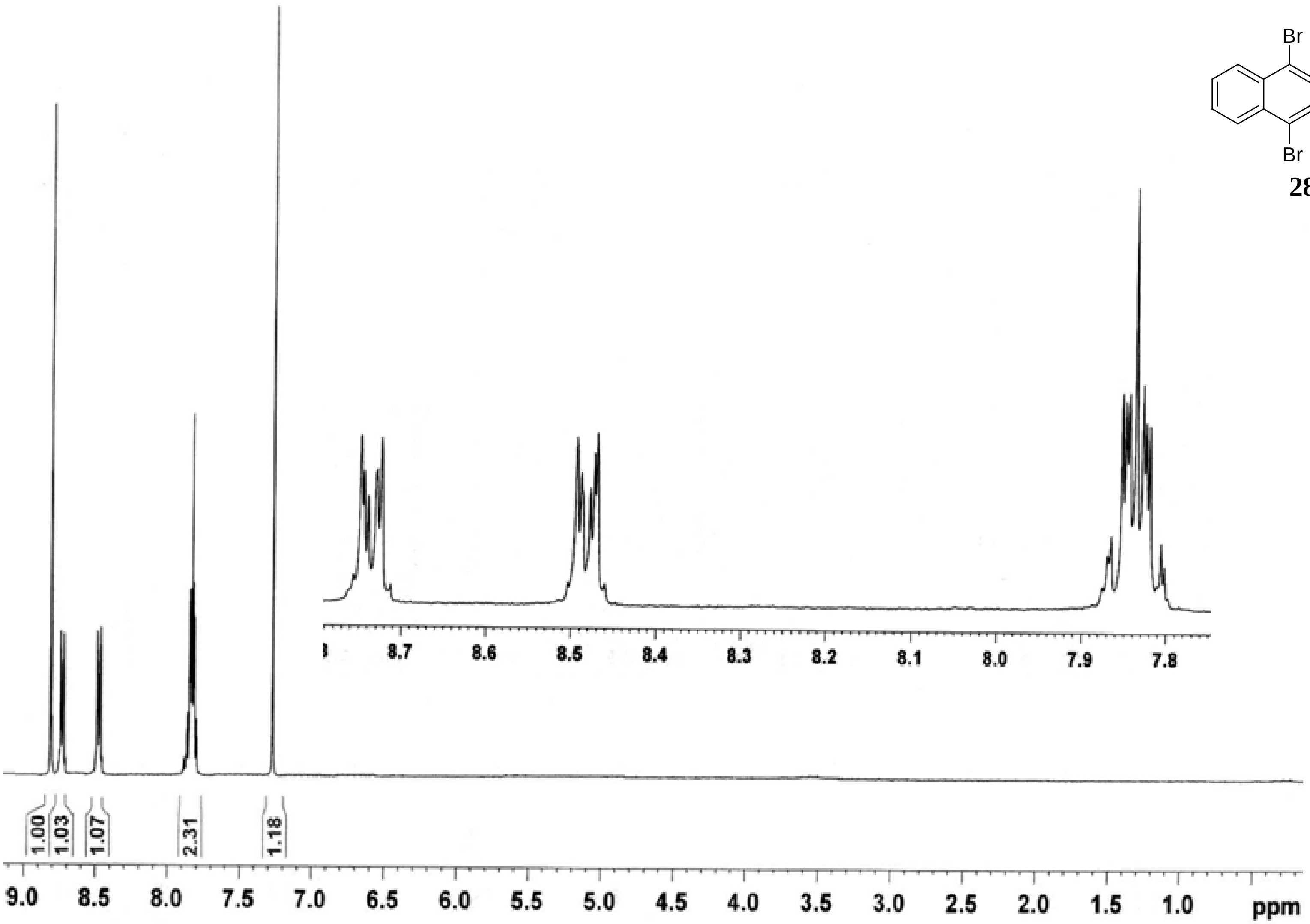
8.7
8.6
8.5
8.4
8.3
8.2
8.1
8.0
7.9
7.8
1.00
1.03
1.07
2.31
1.18
9.0
8.5
8.0
7.5
7.0
6.5
6.0
5.5
5.0
4.5
4.0
3.5
3.0
2.5
2.0
1.5
1.0
ppm

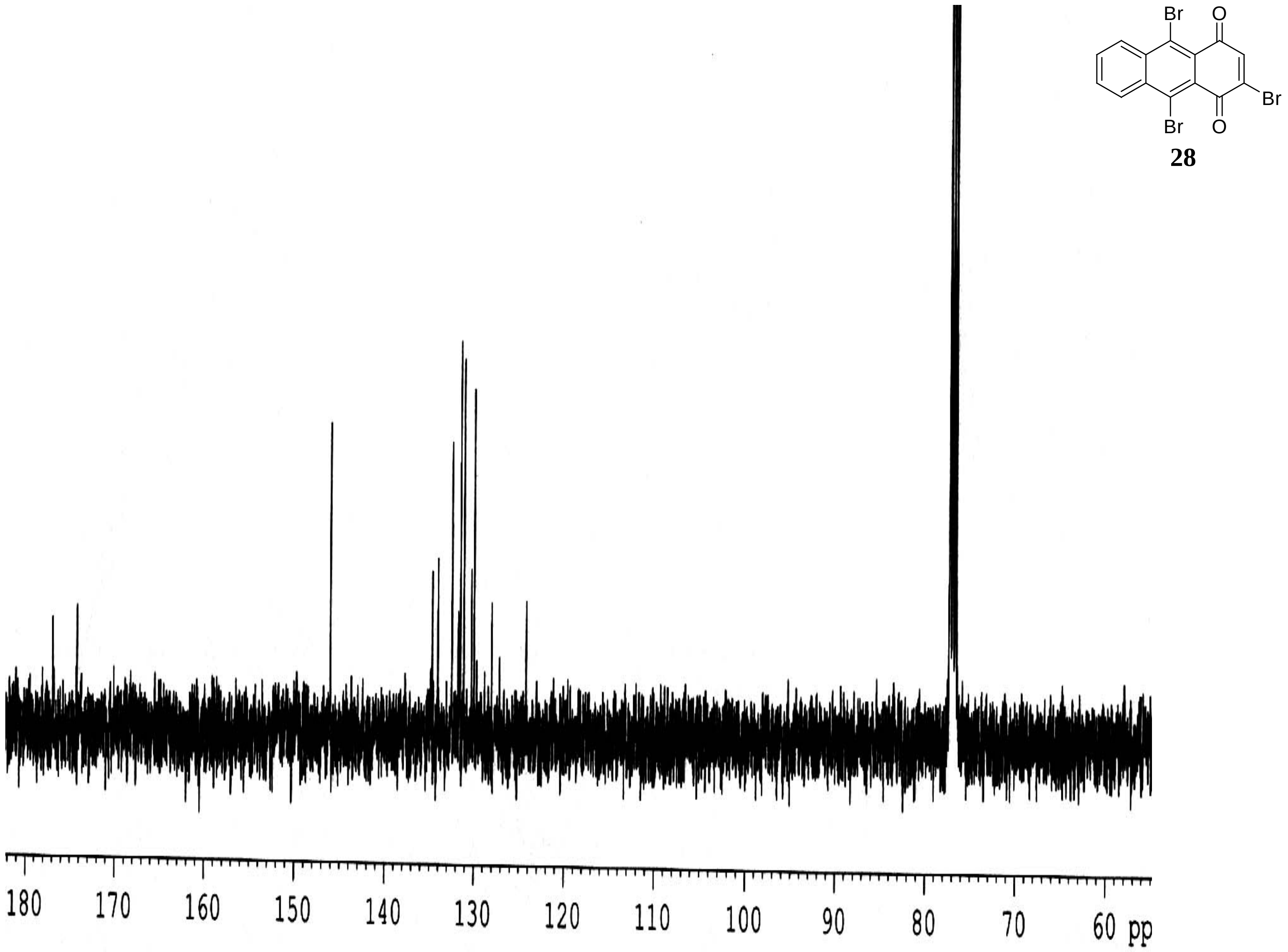

28
